# Photoacoustic Imaging for Liver Disease: The Systems and the Molecules

**DOI:** 10.3390/bios16090497

**Published:** 2026-09-05

**Authors:** Bowen Jiang, Zhixian Lin, Xiaoquan Yang

**Affiliations:** 1 School of Sino-German Robotics, Shenzhen University of Information Technology, Shenzhen 518172, China; jiangbowen@suit-sz.edu.cn; 2 State Key Laboratory of Digital Medical Engineering, Key Laboratory of Biomedical Engineering of Hainan Province, School of Biomedical Engineering, Hainan University, Sanya 572025, China; zxlin@hainanu.edu.cn; 3 MOE Key Laboratory for Biomedical Photonics, Wuhan National Laboratory for Optoelectronics, Huazhong University of Science and Technology, Wuhan 430074, China

**Keywords:** photoacoustic imaging, liver disease, photoacoustic computed tomography, activatable probes, image reconstruction

## Abstract

Liver disease represents a significant global health burden, and its effective management relies on early, accurate diagnosis. Established assessments for liver diseases, ranging from invasive biopsies to conventional noninvasive imaging (e.g., ultrasound, MRI, and CT), are often limited by inadequate specificity, potential safety risks, or unsuitability for dynamic tracking. Photoacoustic imaging (PAI), a hybrid modality that combines optical absorption contrast with deep ultrasonic detection, offers an attractive solution for noninvasive, high-sensitivity, and high-specificity imaging in deep organs such as the liver. This review summarizes recent advances in photoacoustic imaging for liver pathophysiology, beginning with the evolution of imaging systems and extending to the diverse molecules employed for preclinical studies and early clinical trials. Specifically, novel reconstruction algorithms improved the spatial resolution and acquisition speed by up to threefold, Monte Carlo-based fluence compensation increased the deep-tissue signal-to-background ratio by approximately 50%, and a 7-azaindole-modified probe exhibited one-magnitude-higher superoxide-triggered activation than conventional hemicyanine dyes. Furthermore, we discuss key challenges and future perspectives, highlighting the translational potential of PAI as an emerging liver imaging modality.

## 1. Introduction

Liver disease constitutes a substantial and escalating global health challenge, accounting for over 2 million deaths annually (4% of all mortality) [1]. Drawing upon the most current global epidemiological estimates available from the Global Burden of Disease (GBD) 2023 study [2] and the World Health Organization (WHO) 2026 Global Hepatitis Report [3], major contributors include viral hepatitis and alcohol-associated liver disease (ALD), which link to approximately 1.3 million and 0.4 million annual deaths, respectively. Liver cancer was the third-leading cause of cancer death in 2024 (approximately 758,000 deaths) according to the most current GLOBOCAN (Global Cancer Observatory) 2024 data [4], while the total deaths related to metabolic dysfunction-associated steatotic liver disease (MASLD) were estimated to be around 131,000 in 2023 [2]. As to morbidity, acute liver failure (ALF) is relatively rare, with a global incidence estimated to be a few per million [1,5], while the global prevalence of chronic liver disease (CLD) reached more than 1.6 billion cases annually, with an age-standardized incidence rate that continues to climb, particularly among young adults [2,6]. MASLD has emerged as the dominant driver of the global burden, affecting more than one-third of the global adult population and accounting for over 80% of incident CLD cases [2,7,8]. Concurrently, ALD remains the leading cause of cirrhosis in many regions and is exhibiting rising incidence trends [2]. With projections indicating that the incidence of CLD will continue to increase through 2050 [6], primarily fueled by the rising prevalence of metabolic risk factors and alcohol consumption, liver disease poses a critical and expanding threat to global public health systems.

The causes of ALF include viral hepatitis and drug-induced liver injury (DILI) [9,10], although a substantial proportion remains unknown [11]. Chronic liver disease may result from varied viral, metabolic, toxic, or autoimmune factors [12], but its progression typically follows a shared pathological trajectory, advancing from initial hepatocyte injury and inflammation to fibrosis, and ultimately culminating in end-stage complications such as cirrhosis, acute-on-chronic liver failure, and hepatocellular carcinoma (HCC) [13]. This pathological evolution is often insidious: early stages of liver injury are frequently asymptomatic and reversible, whereas the transition to decompensated cirrhosis represents a critical turning point associated with high mortality and limited therapeutic options beyond transplantation.

Consequently, the clinical outcome is heavily dependent on the ability to detect and stage liver injury at an early, modifiable phase. However, accurate diagnosis remains a significant challenge. Liver biopsy, the traditional gold standard, is invasive and unsuitable for large-scale screening, while existing noninvasive biomarkers often lack the sensitivity required to distinguish early-stage fibrosis or specific inflammatory phenotypes. Therefore, the development of precise, noninvasive diagnostic strategies is urgently needed to bridge the gap between rising disease prevalence and the necessity for timely, targeted therapeutic interventions.

### 1.1. Clinical Imaging Examination

With the exception of viral hepatitis and ALI, which can be diagnosed through serological testing, imaging characteristics serve as essential diagnostic criteria for all other liver diseases [14]. Clinical imaging examinations involved in this process include ultrasonography, computed tomography (CT), magnetic resonance imaging (MRI), transient elastography, positron emission tomography (PET)/CT, and single-photon emission computed tomography (SPECT)/CT [14,15,16,17].

Each of these modalities offers distinct advantages, but also possesses inherent limitations for liver disease management. Ultrasonography and transient elastography are widely adopted as first-line tools due to their real-time capabilities, cost-effectiveness, and non-ionizing nature. However, they are highly operator-dependent and lack sufficient soft-tissue contrast for detecting early-stage microscopic lesions. CT provides rapid, high-resolution anatomical mapping, but its routine use is hindered by ionizing radiation and the potential nephrotoxicity of contrast agents. Conversely, MRI and newly developed MRI–PDFF (magnetic resonance imaging–proton density fat fraction) excel in delivering superior soft-tissue contrast and detailed structural visualization without radiation, yet they are significantly limited by high costs, long acquisition times, and susceptibility to respiratory motion artifacts. Furthermore, while functional modalities like PET/CT and SPECT/CT provide valuable metabolic insights, they involve radioactive tracers and exhibit relatively poor spatial resolution. Therefore, there remains a critical clinical need for a comprehensive, integrated solution that incorporates an imaging modality combining deep penetration, non-ionizing radiation, high spatiotemporal resolution, and rich molecular contrast. Photoacoustic imaging, which uniquely brings together all of these capabilities, is well suited to serve as a key component of such a platform.

### 1.2. Photoacoustic Imaging Fundamentals

Photoacoustic imaging (PAI) was developed based on the photoacoustic effect. Generally, when an absorber is illuminated by a relative short-pulse light, the absorbed optical energy would be partially converted into heat and introduce local thermoelastic expansion, which ultimately generates acoustic waves [18,19,20]. With the light intensity fall in the linear excitation zone, the detected acoustic signal will be proportional to the percentage of absorbed light converted into heat, the optical absorption coefficient, and the local optical fluence, providing the opportunity to reconstruct the optical absorption map within the tissue.

PAI can be set up in two major modalities, namely photoacoustic microscopy (PAM) and photoacoustic tomography (PAT) [21]. PAM employs focused optical excitation or ultrasonic detection to achieve micrometer-level spatial resolution at millimeter imaging depths, enabling the assessment of fine structures such as microvasculature and collagen fibers during liver disease progression [22,23,24]. PAT, on the other hand, offers submillimeter resolution at centimeter-scale depth with wide-field rapid scanning, which is ideal for whole-organ evaluation or even the whole-body imaging of small animals [25,26]. In the context of liver imaging, the two modalities are therefore complementary rather than competing. PAM is preferred when the goal is to resolve and quantify microvascular changes, lipid accumulation, or collagen deposition at high spatial resolution, whereas PAT is better suited for whole-organ anatomical and functional evaluation, as well as longitudinal monitoring of disease progression in preclinical models.

In PAM, the optical and ultrasonic foci are generally configured in a confocal alignment to obtain high spatial resolution and sensitivity, and thus the lateral resolution, which is determined by the sharper focus, further classifies PAM into optical-resolution PAM (OR-PAM) and acoustic-resolution PAM (AR-PAM) [24,27,28]. Image formation in PAM typically relies on a point-by-point scanning mechanism. Upon each laser pulse, a time-resolved photoacoustic signal is recorded to form a one-dimensional depth profile, commonly referred to as an A-scan. By mechanically or optically scanning along a linear path, a two-dimensional cross-sectional image, or B-scan, is acquired. Ultimately, a complete 3D volumetric image is rendered by performing a two-dimensional raster scan over the region of interest.

**Figure 1:**
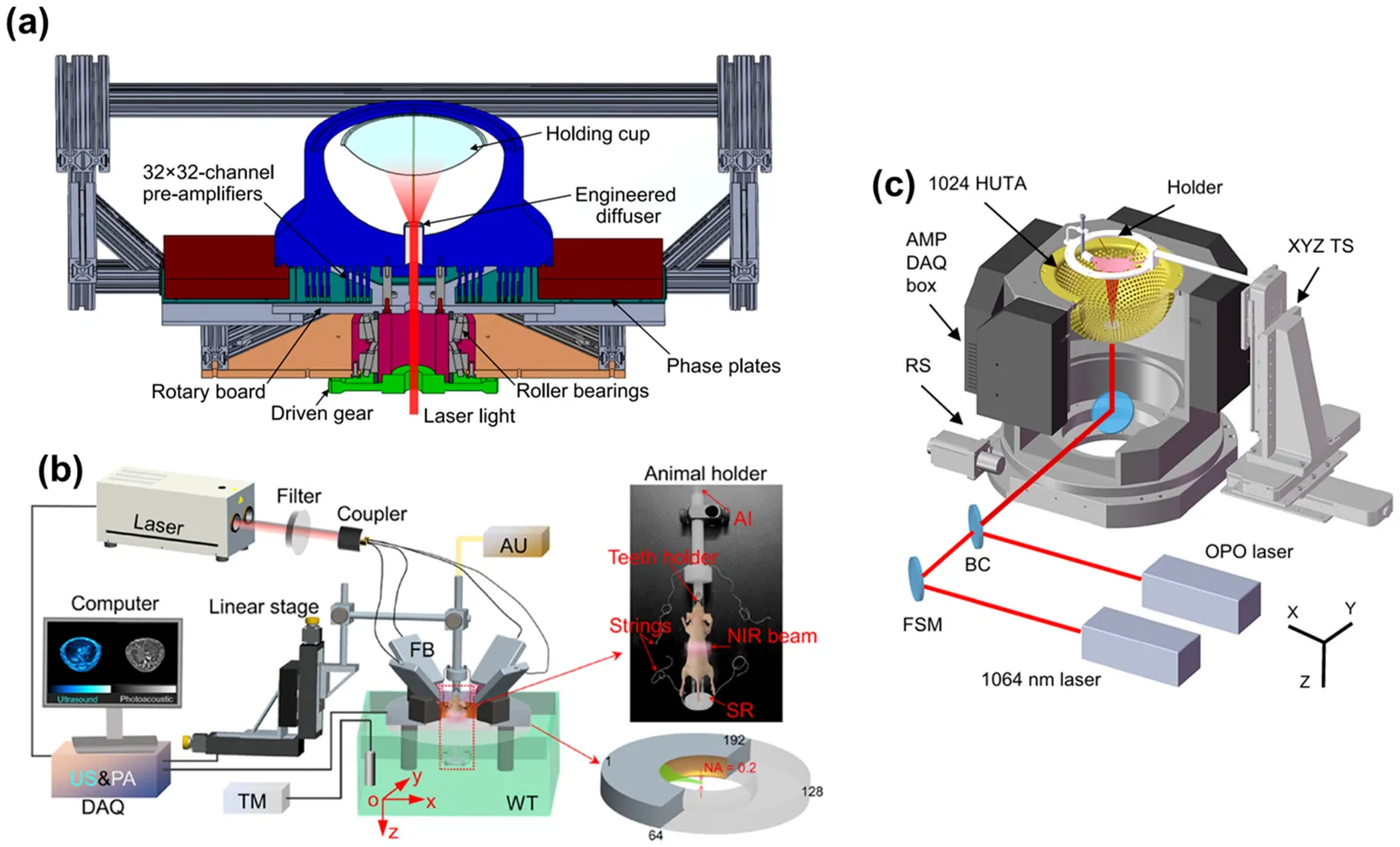
Three major configurations of photoacoustic computed tomography (PACT) systems. (**a**) Arc-shaped transducer array PACT system. Reproduced with permission from [32]. Copyright 2021, the authors, published by Springer Nature under CC BY 4.0 license. (**b**) Full ring-shaped transducer array PACT system. Reproduced with permission from [34]. Copyright 2022, the authors, published by Optica Publishing Group under the terms of the Optica Open Access Publishing Agreement. (**c**) Hemispherical transducer array PACT system. Reproduced with permission from [36]. Copyright 2025, the authors, published by Springer Nature under CC BY 4.0 license.

Typical arrangement of the PAT system is much more like CT: excitation light illuminates the tissue, while ultrasonic transducer collects the acoustic signal with multiple angles [29,30]. The ultrasonic transducer may be a rectangular [31], arc-shaped [32], or ring-shaped array [33,34], or even a single-element one that detects widefield PA signals encoded by ergodic relay [35]. Since volumetric imaging capability is directly dictated by transducer geometry, PAT systems can be categorized into arc-shaped (including rectangular), ring-shaped, and hemispherical array [36] configurations, as illustrated in Figure 1. Rectangular and arc-shaped arrays, with only tens of elements, offer limited angular coverage. Full-space 3D imaging therefore requires mechanical rotation and inherently suffers from limited-view artifacts. Ring-shaped arrays, composed of hundreds of elements arranged in a closed circle, capture complete cross-sectional tomograms per laser pulse, and volumetric imaging is achieved solely by linear translation of the object. Hemispherical arrays go further, distributing hundreds to over a thousand elements across a spherical surface to realize near-complete solid-angle sampling with a single pulse. This enables single-shot, high-fidelity isotropic 3D imaging without any mechanical scanning, fundamentally eliminating the limited-view artifacts that compromise arc and ring platforms.

In contrast to PAM, computational processing must be applied to raw acoustic data to reconstruct the absorption distribution in PAT. Conventional methods, such as direct back projection [37,38], construct mathematical models under idealized conditions to recover tissue structure efficiently. However, image quality degrades significantly under sparse sampling, and the typical assumptions of a uniform acoustic speed fail to account for inherent acoustic heterogeneity. While model-based iterative reconstruction accommodates acoustic velocity variations for more accurate reconstructions, it incurs substantial computational complexity, heavily limiting real-time imaging applications. Recently, learning-based methods have emerged as powerful alternatives. Rooted in machine learning, these approaches map raw acoustic signals to target structures via deep neural networks to suppress artifacts and enhance signal-to-noise ratios [39,40,41,42].

## 2. Photoacoustic Liver Imaging Systems

### 2.1. Photoacoustic Tomography (PAT) for Deep Liver Evaluation

In the context of liver imaging, PAT holds particular promise for assessing hepatic steatosis, tumor angiogenesis, hemodynamics, and treatment response, while conventional PAT systems have yet to fully harness the technology’s imaging potential. Over the last several years, remarkable progress in PAT system architecture, image reconstruction algorithms, and multimodal integration have been witnessed, accelerating the process of preclinical research and clinical translation.

**Figure 2:**
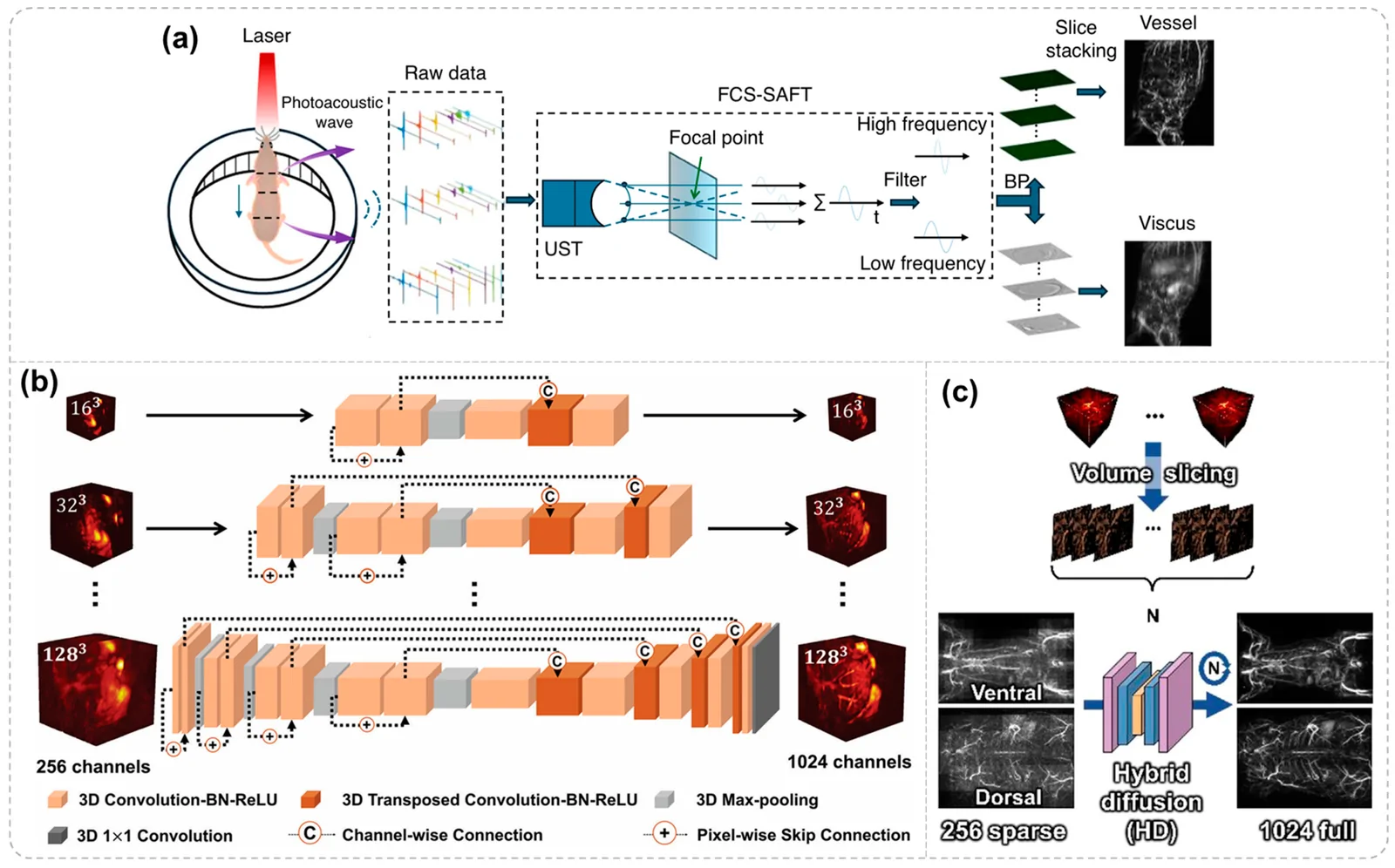
Advanced computational reconstruction and enhancement strategies for photoacoustic computed tomography (PACT). (**a**) The signal processing workflow of frequency component selection-based synthetic aperture focusing technology (FCS-SAFT) used in the SRCPT system. Reproduced with permission from [43]. Copyright 2024, the authors, published by Springer Nature under CC BY 4.0 license. (**b**) The 3D progressive U-net (3D-pU-net) architecture utilized in the DL-PACT framework. Reproduced with permission from [49]. Copyright 2023, the authors, published by Wiley-VCH GmbH under CC BY 4.0 license. (**c**) The Hybrid Diffusion (HD) model architecture employed in the HD-PACT framework. Reproduced with permission from [50]. Copyright 2026, the authors, published by Wiley-VCH GmbH under CC BY 4.0 license.

Conventional PAT systems suffer heavily from limited volumetric coverage and anisotropic resolution. By coupling a tunable nanosecond pulsed laser with a high-frequency ultrasound transducer array and introducing frequency component selection-based synthetic aperture focusing technology (FCS-SAFT) for coherent superposition of ultrasonic echoes, Lv et al. constructed a spatiotemporally resolved clearance pathway tracking (SRCPT) PAT system [43]. The system achieved a large field of view (FOV), covering the entire mouse body while maintaining uniform high resolution in 3D space (Figure 2a). Furthermore, they utilized a Monte Carlo model to calibrate the light fluence distribution in deep tissues, eliminating quantitative errors caused by optical attenuation.

Emerging three-dimensional photoacoustic computed tomography (3D-PACT) enables high-speed volumetric imaging with superior image quality, deep penetration, and isotropic spatial resolution [32,44,45] by advanced system design and engineering. Kim et al. developed a high-definition 3D multiparametric PACT system based on a 1024-element hemispherical ultrasound transducer array [46], achieving noninvasive monitoring of angiogenesis, carcinogenesis, hypoxia, and pharmacokinetics in metastatic liver tumor models in living mice, with imaging time within an effective volumetric FOV of 12.8 × 12.8 × 12.8 mm^3^ less than 0.3 s. Tong et al. implemented a time-gated strategy to address the challenge of respiratory motion artifacts during imaging [47], and quantified statistically significant differences in multiple PAT-derived features between lean and obese rat livers, providing a valuable method for preclinical evaluation of non-alcoholic fatty liver disease (NAFLD). Wang et al. introduced a spatiotemporal-integration (STINT) method to physically augment detection density by rotating a Fibonacci grid transducer array at small angular increments [36]. This synthesizes an equivalent high-density detection array to reduce artifacts caused by spatial undersampling. Coupled with dual-speed-of-sound universal back projection (UBP) to mitigate acoustic inhomogeneity, the developed 3D-PanoPACT system holds an exceptionally broad 3D FOV while preserving high spatiotemporal resolution. The system captured the entire hepatic arterial network and correlated it with cerebrovascular functional dynamics, offering more accurate quantification of liver perfusion and vascular morphology, and furthermore unprecedented insights into inter-organ cross talk and systemic hemodynamic responses. More recently, Wang et al. introduced three-dimensional aperture-driven photoacoustic tomography (3D-ADPAT) [48] by synergistically combining a high–numerical-aperture focused ultrasonic transducer (HNA-FUT) with a phase-inverted focusing-equivalent reconstruction (PIFER) algorithm, yielding an approximately 2.21-fold improvement in spatial resolution and an approximately 10-fold increase in contrast-to-noise ratio compared with conventional quasi-point-transducer PACT. Using a 3.5 MHz HNA-FUT and dual-wavelength (1064/532 nm) excitation at a 10 Hz repetition rate, the system achieved an axial resolution of ~170 µm and a lateral resolution of ~420 µm, and resolved deep-seated anatomies and tracked the small-molecule (A1094) metabolic pathway in the liver during in vivo whole-body imaging. A barrel-shaped array implementation comprising 640 HNA-FUT elements was further designed to support dynamic imaging over a large field of view or across variable spatiotemporal scales, illustrating a scalable route toward high-speed, large-FOV 3D photoacoustic systems for organ-level and translational applications.

An alternative approach to break the trade-off between dense detection array and system cost is the deep-learning method. The transformative framework enables high-quality image reconstruction from sparsely sampled or limited-view data. Choi et al. developed a deep learning-enhanced multiparametric dynamic volumetric PACT (DL-PACT) system that qualitatively and quantitatively diminish limited-view artifacts while improving temporal resolution [49]. They employed a three-dimensional progressive U-shaped enhancement network (3D-pU-net) to directly map sub-sampled “cluster-view” data to high-resolution “full-view” targets (Figure 2b). To preserve intricate biological structures, the network is iteratively optimized using a hybrid loss function that combines voxel-wise L1 loss with 3D structural similarity (3D-SSIM). DL-PACT requires only approximately a quarter of the transducer elements in a conventional hemispherical array, significantly reducing system cost and complexity while maintaining the volumetric and multiparametric imaging capabilities essential for hepatic research.

Pushing the boundaries of sparse sampling even further, Jeong et al. introduced a hybrid diffusion model for PACT (HD-PACT) [50], specifically designed to enhance 3D multiparametric imaging under extreme hardware constraints (e.g., utilizing only 128 or 256 out of 1024 elements) (Figure 2c). HD-PACT leverages a PA-optimized Gabor filter in its forward diffusion process to accurately model and simulate the complex radial-streak noise typical of sparse acoustic arrays. During the reverse generative denoising process, it utilizes an innovative efficient-hybrid mamba (EHM) module. This module seamlessly integrates efficient channel attention (ECA), efficient self-attention (ESA), and mamba state space models to extract crucial long-range spatial dependencies with exceptional computational efficiency. Furthermore, by incorporating an error-modulated model (EMM) for time-embedding alignment and exploiting 3D contextual information from adjacent image slices, HD-PACT successfully reconstructed dynamic hypoxia, pharmacokinetics, and angiogenesis during tumor progression. This finding demonstrates that advanced generative diffusion models can recover high-fidelity functional and structural dynamics even on low-end PACT configurations. The performance of SRCPT, 3D-PAT, DL-PACT, 3D-multiparametric PACT, and HD-PACT are compared in Table 1.

For the deep-learning method, acquiring perfectly paired, high-quality in vivo ground-truth datasets for training supervised networks remains practically challenging. To address this, self-supervised and unsupervised learning frameworks, such as Noise2Noise, Noise2Void, and zero-shot learning, offer robust paradigms for in vivo image denoising and sparse reconstruction without relying on pristine paired data [51,52,53,54]. By integrating these advanced computational priors, deep learning-empowered PACT systems can achieve superior reconstruction performance in challenging deep-tissue and limited-angle scenarios, bridging the gap between hardware limitations and clinical diagnostic needs.

While deep learning yields high-quality images at low cost, concerns remain regarding model generalizability, dependence on training data, and quantitative bias. In DL-PACT, the network was trained on 1089 volumetric datasets from 18 rats, using full-view reconstructions from the same system as reference targets. It therefore learned a mapping specific to that acquisition configuration rather than an independent physiological ground truth, limiting generalization when detector geometry, tissue properties, noise, or imaging conditions differ substantially from the training set. For HD-PACT, although trained on 900 nm PA data from rat ventral and dorsal planes, it retained useful structural information when tested on sagittal-plane data, other wavelengths, tumor-bearing mice, and human palm data. However, this does not equate to clinical validation for liver PAI, as palm data do not reproduce the optical attenuation, acoustic heterogeneity, respiratory motion, abdominal wall thickness, or strong hemoglobin background encountered in hepatic imaging. More importantly, deep-learning reconstruction can affect quantitative functional imaging. HD-PACT enabled spectral unmixing and estimation of total hemoglobin (HbT) and oxygen saturation (sO_2_), but the authors reported overestimation of reduced sO_2_ in tumors, indicating that structural image quality does not guarantee quantitative accuracy. Since HbT and sO_2_ are derived from wavelength-dependent PA amplitudes, any learned amplitude distortion or wavelength-dependent bias may propagate into spectral unmixing and alter physiological estimates. Therefore, deep-learning reconstruction should be evaluated not only by image-quality metrics such as Peak Signal-to-Noise Ratio (PSNR) and SSIM but also by signal-amplitude preservation, spectral fidelity, quantitative bias, uncertainty, and agreement with independent physiological or histological references.

Dual-mode integrated PAT and US systems are particularly attractive for clinical translation, as they can be implemented on existing ultrasound platforms. Lafci et al. developed a transmission reflection optoacoustic ultrasound (TROPUS) system for quantitative multimodal assessment of NAFLD [55]. The system provides complementary information about liver structure, mechanical properties, and molecular composition. Godfrey et al. presented a combined system for simultaneous 3D multispectral photoacoustic fluctuation imaging (MS-PAFI) and ultrasound Doppler [56]. Complementary label-free 3D images of blood oxygenation (via quantitative PAI) and blood-flow dynamics (via ultrasound Doppler) hold great promise for comprehensive liver vascular assessment. More recently, ultrafast ultrasound and ultrasound localization microscopy (ULM) have provided a further opportunity to increase the temporal and spatial information available from multimodal PA/US imaging. Zhao et al. developed an interleaved photoacoustic and fast super-resolution ultrasound imaging framework that combined PA oxygenation measurements with high-resolution vascular flow mapping [57], achieving dual-modal imaging at less than 2 s per frame in vivo. The approach used sparsity-constrained optimization to accelerate ultrasound localization imaging, thereby reducing the acquisition-speed mismatch between PA and super-resolution ultrasound. Related work has further demonstrated integrated ultrafast ultrasound and PA acquisition for multiparametric hemodynamic imaging [58]. These developments suggest that ultrafast ultrasound/fUS-assisted PAI could provide complementary measurements of oxygenation, blood-flow dynamics, and vascular architecture, potentially improving the characterization of hepatic perfusion and vascular remodeling. However, most current demonstrations have focused on brain, kidney, or other preclinical models rather than the human liver, and the feasibility of applying these high-temporal-resolution strategies to hepatic imaging remains to be evaluated.

Several conventional PAT systems have already been successfully translated into commercial products for preclinical and clinical liver research. Notable representatives include the multispectral optoacoustic tomography (MSOT) inVision system (iTheraMedical, GmbH) [59,60], the Vevo LAZR system (FUJIFILM, VisualSonics) [61,62], the laser optoacoustic imaging system—3D (LOIS-3D, TomoWave Laboratories) [63], and the single-impulse panoramic photoacoustic computed tomography system (SIP-PACT, Union Photoacoustic Technologies) [64]. A comparative overview of their core technical parameters is summarized in Table 2. Overall, MSOT systems acquire images at multiple wavelengths and perform spectral unmixing to quantify the concentrations of different tissue chromophores, while Vevo LAZR is a dual-modality preclinical imaging platform that integrates high-frequency ultrasound with photoacoustic technology, providing inherent high-resolution co-registration of anatomical, functional, physiological, and molecular characterization of small-animal models. LOIS-3D offers an ultrahigh optical absorption sensitivity of 0.03 cm^−1^ and a tunable near-infrared broadband wavelength range of 650–2300 nm that enables deep-tissue imaging. SIP-PACT provides motion artifact-free real-time dynamic volumetric imaging with a maximum FOV of 48 mm diameter, 100 mm axial scanning length.

**Figure 3:**
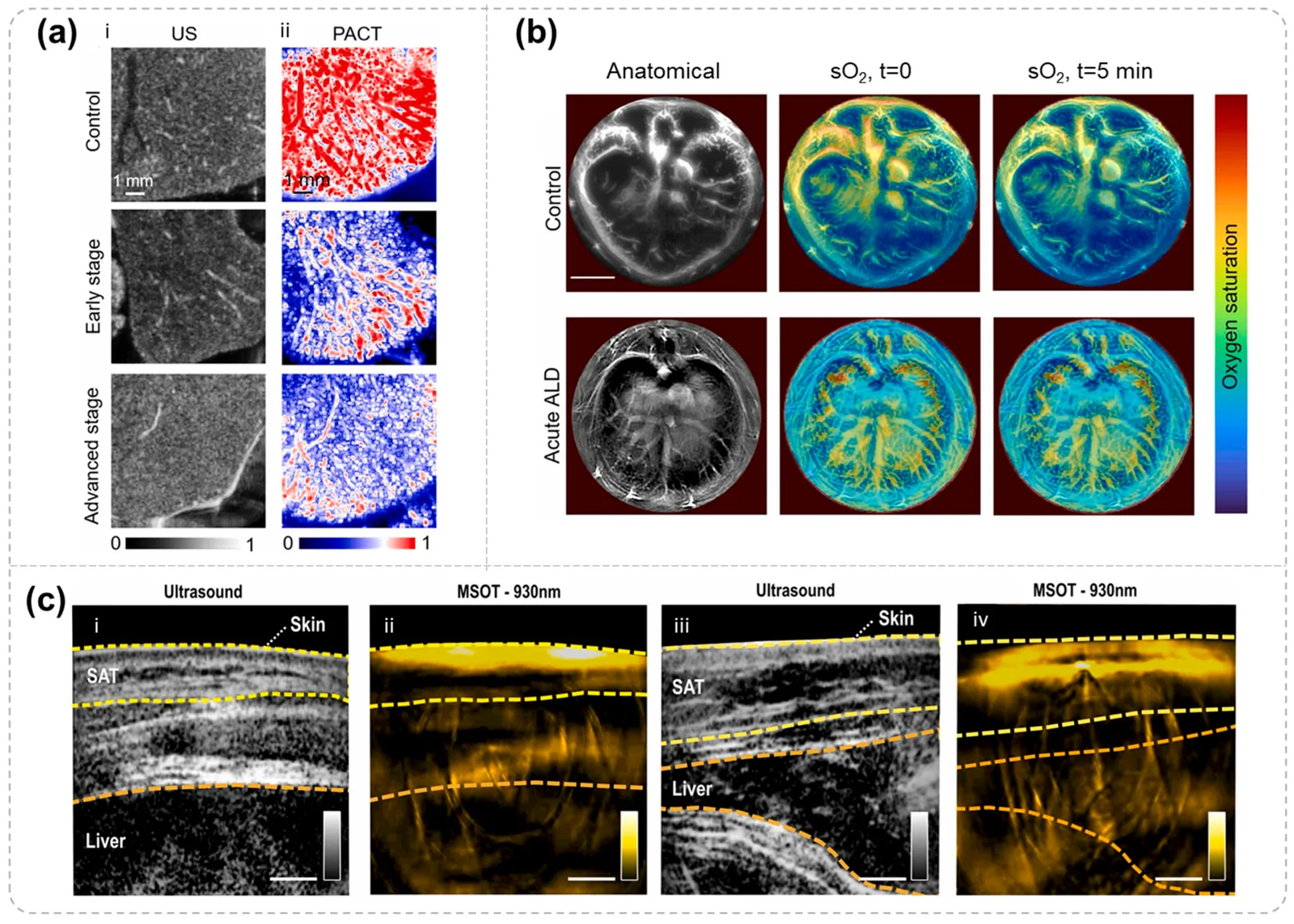
Representative structural and functional PA images obtained by preclinical PACT systems. (**a**) Transverse (**i**) US and (**ii**) PA images of mouse liver with hereditary tyrosinemia type 1 (HT1) at different stages acquired by Vevo LAZR system. Adapted from [61] under CC BY 4.0. (**b**) SIP-PACT functional assessment: Anatomical and sO2 images of mouse livers from control and acute ALD groups. Scale bar: 5 mm. Adapted from [76] under CC BY 4.0. (**c**) Human hand-held MSOT imaging: (**i**) US image and (**ii**) MSOT image recorded at 930  nm of the hypochondriac region of a healthy volunteer. (**iii**) US image and (**iv**) 930  nm-MSOT image of the same region of a patient with liver steatosis. Adapted from [70] under CC BY 4.0.

Based on the Vevo system, a novel data-driven superpixel photoacoustic unmixing (SPAX) framework was adapted for liver fibrosis detection by Sultan et al. [75]. Instead of pre-knowing the expected chromophore spectra, SPAX models the light fluence distribution to compensate for spectral coloring. Quantification accuracy of collagen is improved and a significant correlation between increased collagen photoacoustic signal intensity and advanced fibrosis stages is revealed. Beyond serving as a validation platform for algorithmic development, these imaging systems also offer a robust and stable platform for longitudinal monitoring. As demonstrated in Figure 3a and Figure 3b, the Vevo LAZR and SIP-PACT systems distinctly capture the progressive disruption of hepatic vasculature in a hereditary tyrosinemia type-1 (HT1) mouse model [61] and a marked decrease in sO_2_ in mice with acute ALD [76], respectively.

These commercial systems also enable researchers to explore the clinical feasibility of PAT. As shown in Figure 3c, by analyzing the spectral region around 930 nm, where lipids exhibit characteristic absorption, Fasoula et al. applied MSOT to measure liver and surrounding tissues in five patients with liver steatosis and five healthy volunteers [70]. The results revealed significantly higher absorption at 930 nm in the patient group, while no significant differences were observed for the control group.

### 2.2. Photoacoustic Microscopy (PAM) for Hepatic Microenvironment

**Figure 4:**
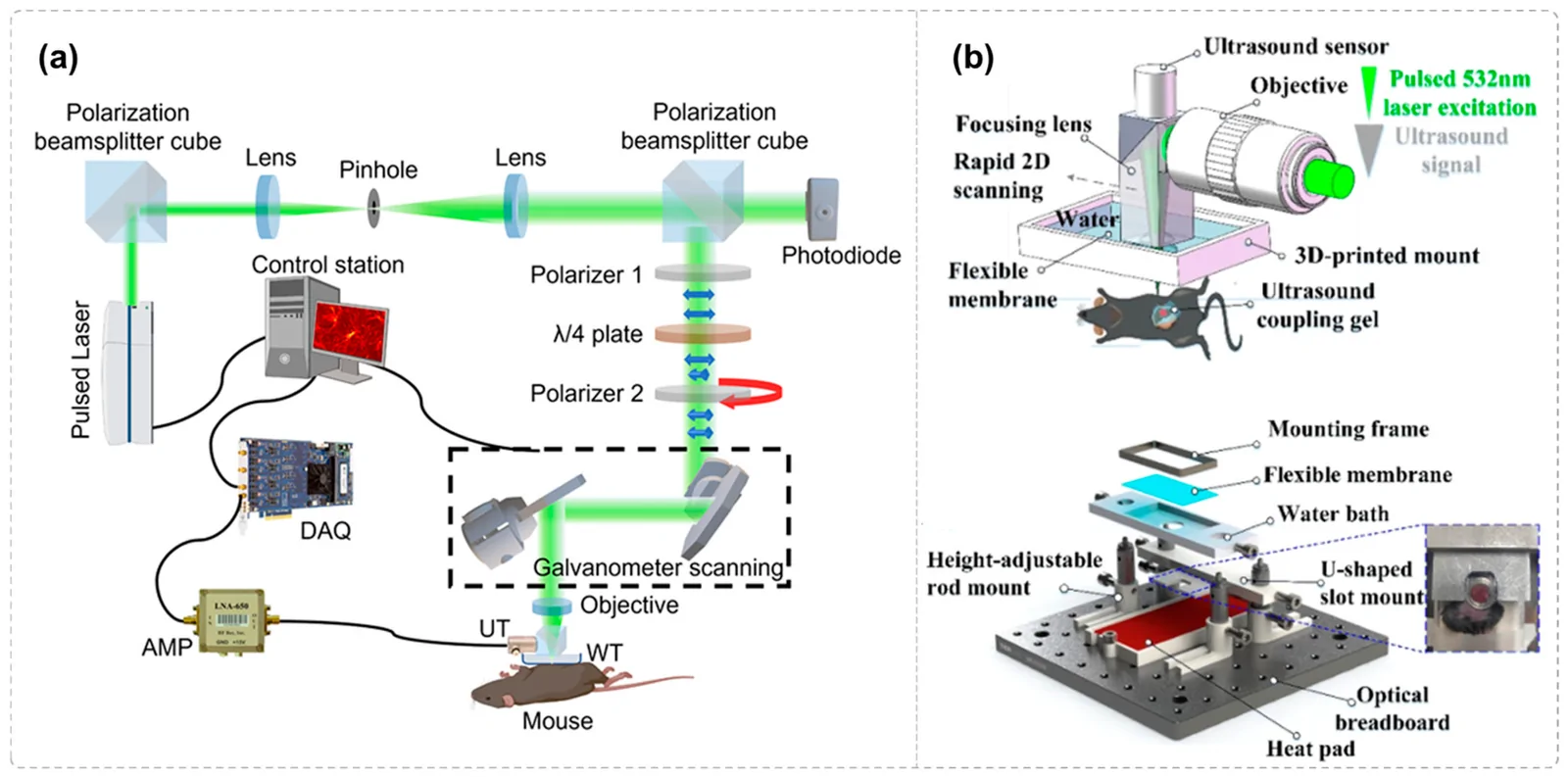
Schematic illustrations of advanced photoacoustic microscopy (PAM) configurations for hepatic microenvironmental assessment. (**a**) The linear dichroism photoacoustic microscopy (LD-PAM) system. This specialized setup incorporates polarization-modulating optical elements to noninvasively assess and quantify the dichroic properties of collagen fiber deposition. DAQ, digitizer acquisition; AMP, amplifier; UT, ultrasound transducer; WT, water tank. Reproduced with permission from [77]. Copyright 2025, the authors, published by Elsevier GmbH under CC BY license. (**b**) The optical-resolution photoacoustic microscopy (OR-PAM) system with a custom 3D-printed liver imaging mount. Reproduced with permission from [81]. Copyright 2025, the authors, published by Elsevier GmbH under CC BY license.

Given that the microscopic mechanisms underlying liver disease progression and immune responses remain largely unclear, PAM has attracted significant attention in the field of liver imaging due to its unique capabilities in visualizing the hepatic microvasculature, quantifying lipid accumulation, monitoring oxygen metabolism, and assessing liver function without the need for exogenous contrast agents. Although hemoglobin provides strong contrast for visualizing the microvasculature, it generates substantial background signal in liver fibrosis evaluation, where collagen fibers are the signal of interest. To suppress this blood-derived background, a novel linear dichroism photoacoustic microscopy (LDPAM) technique was developed [77] (Figure 4a). In LDPAM, PA images are acquired under polarized excitation at four different orientations, from which the collagen fiber degree of dichroism (CDOD) is calculated. CDOD has been shown to correlate strongly with the collagen proportional area (CPA) score, demonstrating the method’s capability for noninvasive and effective staging of liver fibrosis. However, it should be noted that this polarization-based approach has so far been validated primarily in small-animal models. In human liver, strong optical scattering in the overlying tissues would likely randomize the polarization state, limiting its immediate clinical applicability.

A complete understanding of dynamic microenvironmental processes relies on long-term, continuous monitoring. Intravital fluorescence/photoacoustic imaging of immune activity in tumor–liver metastases for over 10 days was achieved by implementing a drawer-type abdominal window with an acrylic/resin coverslip by Deng et al. [78]. The window drastically minimizes motion artifacts induced by respiration and heartbeat, enabling the acquisition of stable, high-resolution images without the need for adhesives that may induce inflammatory responses. The design is further elevated by using an ultra-thin polymethyl methacrylate coverslip along with a custom 3D-printed liver imaging mount (Figure 4b) [79], extending the long-term imaging ability to over six weeks. Continuous in vivo monitoring of microvascular changes in a non-alcoholic steatohepatitis (NASH) mouse model was successfully performed, and for the first time, separation of hepatic sinusoids and vessels in PAM images were achieved, improving the accuracy of 3D spatial characterization of microvasculature in preclinical liver disease research.

On the commercial front, the InnoLaser G2 stands out as a high-performance multimodal optical-resolution photoacoustic microscopy (OR-PAM) platform [80] for probing the microscopic environment. It features an ultrahigh lateral resolution of < 2 μm (sub-micron resolution optional) and native co-registered multimodal imaging capabilities, with optional integrated fluorescence (FL), bright-field reflection, and optical coherence tomography (OCT) modules, enabling label-free quantitative analysis of HbT, sO_2_, blood-flow velocity, and vascular morphology with high stability for longitudinal in vivo studies. Li and Lv et al. effectively visualized hepatic sinusoids and quantified vascular structural and functional changes in a mouse model of liver fibrosis using the G2 PAM system [81], and introduced lacunarity, Evans blue permeability, and blood oxygen saturation as PAM-based quantitative parameters of liver function and integrity.

## 3. Application of Exogenous Photoacoustic Contrast Agents in Hepatic Imaging

Hemoglobin exhibits strong absorbance in the visible-light spectrum, while lipids, water, and collagen characteristically absorb in the near-infrared region. These biomolecules serve as key endogenous contrast agents for photoacoustic imaging. Hemoglobin enables structural and functional visualization of the vasculature. The liver, one of the most highly vascularized solid organs (receiving approximately 25% of total cardiac output [82]), is exceptionally rich in blood supply. This inherent abundance of hemoglobin naturally provides a strong endogenous contrast, making photoacoustic imaging an ideal tool for evaluating hepatic hemodynamic alterations, sinusoidal capillarization, and angiogenesis without the need for exogenous dye administration. Furthermore, during the progression of chronic liver diseases, such as hepatic fibrosis and cirrhosis, the pathological remodeling of the microenvironment is characterized by the excessive deposition of extracellular matrix, primarily consisting of collagen fibers. These collagen networks exhibit specific photoacoustic characteristics and unique linear dichroism properties [77]. Consequently, collagen serves as another crucial endogenous contrast source, enabling PAI to directly visualize fibrotic septa and noninvasively stage liver fibrosis based entirely on its intrinsic molecular signatures.

While PA imaging systems continue to achieve higher spatiotemporal resolution in the liver, the molecular specificity provided by endogenous contrasts (hemoglobin, melanin, lipids, etc.) remains inherently limited. The progression of liver diseases (e.g., from inflammation and fibrosis to liver failure or hepatocellular carcinoma) is often accompanied by insidious alterations at the molecular level and within the metabolic microenvironment. Thus, the introduction of exogenous PA contrast agents (including clinically approved dyes and targeted molecular probes) is particularly critical.

### 3.1. Dynamic Monitoring of Liver Function and Clearance Rates Based on Conventional Dyes

Dye-based probes, especially near-infrared dyes, form the cornerstone of PA molecular imaging. Dynamic tracking of dye clearance in the liver enables noninvasive in vivo investigation of pharmacokinetics. For instance, IRDye800CW and mitoxantrone have been used to quantify and visualize the competitive clearance between hepatic and renal excretion routes in mice, establishing a technical foundation for kinetic analysis in precision medicine [43].

Beyond pharmacokinetic tracking, ICG clearance tests further permits clinical evaluation of liver functional reserve (LFR). As hepatic function can decline significantly before structural changes become apparent, LFR assessment offers a vital window into early functional impairment that anatomy-based imaging alone cannot capture. Traditional ICG clearance tests rely on invasive spectrophotometry or pulse dye densitometry (PDD), whose accuracy remains clinically questionable [83]. PA imaging has provided a groundbreaking solution to this dilemma. By employing MSOT to isolate ICG metabolic signals from endogenous backgrounds (e.g., collagen, hemoglobin) within a single field of view, simultaneous mapping of collagen-specific structural remodeling and quantification of LFR impairment during fibrosis progression can be achieved [84]. This approach has now moved toward clinical translation. In healthy volunteers, PA-derived kinetic parameters, including ICG elimination rate constant and half-life, showed a strong positive correlation (r = 0.9649) with the gold-standard in vitro spectrophotometry, with no statistically significant differences [85]. These results overcome the long-standing accuracy limitations of noninvasive PDD.

### 3.2. Smart Activatable Photoacoustic Probes for Molecular Diagnosis

Conventional near-infrared dyes like ICG are intrinsically “always-on” contrast agents, lacking the specificity to recognize pathological biomarkers. In early-stage liver diseases such as DILI, NAFLD, and progressive hepatitis, metabolic and microenvironmental disturbances occur long before structural damage, which passive dye circulation simply cannot capture.

To bridge this gap, smart activatable PA probes have been developed. By equipping chromophore backbones with recognition moieties or cleavable linkers, these probes selectively react with specific biomarkers, triggering profound structural rearrangements, intramolecular charge transfer (ICT), or photoinduced electron transfer (PeT), to produce sharp “off-to-on” signal activations or ratiometric spectral shifts. The ratiometric readout inherently neutralizes errors from light attenuation, heterogeneous probe distribution, and respiratory motion, dramatically suppressing background noise. This enables PAI to dynamically track hepatic pathophysiological evolution at molecular resolution.

#### 3.2.1. Activatable Probes Targeting Hepatic Biomarkers (Enzymes)

One major class of activatable PA probes is enzyme-based. In complex hepatic drug metabolism, the anomalous regulation of basal enzymes is a robust precursor to acute parenchymal toxicity. This has inspired the development of activatable PA probes that leverage enzymatic cleavage to selectively relieve intramolecular quenching or restore ICT. For instance, carboxylesterase (CE), a pivotal detoxification enzyme, drops sharply during acute liver injury. Chen et al. successfully translated gradient CE attenuation into a quantifiable early warning of acetaminophen (APAP)-induced acute liver damage with the dual-modality probe QHD-CE, in which ester bond hydrolysis altered the chromophore’s electronic conjugation [86].

This strategy has been extended to hypoxic stress monitoring. Severe hepatotoxicity often triggers localized microcirculatory disorders that upregulate nitroreductase (NTR). Fan et al. designed the probe NO_2_-CS, which employs an NTR-triggered nitro-to-amino cascade reduction [87]. Triggered exclusively in hypoxic microenvironments, this cascade relieves the ICT blockade and activates a strong PA signal, overcoming the scattering limitations of pure fluorescence imaging. Beyond acute alerts, persistent toxic stimulation drives hepatocytes toward cellular senescence. To noninvasively track this progression, Yan et al. exploited the specific substrate cleavage of beta-galactosidase to construct the probe SGal-HD [88]. This system successfully mapped the spatial distribution of senescent cells in deep tissues, offering an indispensable tool for assessing long-term chemotherapeutic toxicity.

Among hepatic enzymes, gamma-glutamyltransferase (GGT) stands out for its involvement in progressive fibrosis and hepatocellular carcinoma (HCC). Early designs by Wen et al. utilized straightforward GGT-mediated cleavage to turn on PA signals, validating its utility in evaluating hepatoprotective drug efficacy [89]. To accommodate more complex preclinical conditions, Wang et al. optimized the molecular backbone to yield the ETYZE-GGT probe, which captured GGT abnormalities across autoimmune hepatitis, NAFLD, and ultra-early HCC induction stages [90]. However, traditional single-response probes inevitably suffer from background noise caused by non-specific diffusion. To circumvent this, Miao et al. elevated the design to a “dual-lock” architecture, creating the imaging probe IP [91]. This reporter sequentially couples cRGD peptide-mediated targeting of integrins on activated hepatic stellate cells with GGT-specific enzymatic activation. The dual constraint ensures that near-infrared absorption is exclusively turned on within the fibrotic lesion, drastically suppressing non-specific background and representing a major step toward precise theranostics.

To transition from bench to bedside, enzyme-activatable probes must undergo rigorous biosafety evaluations. Encouragingly, recent preclinical studies reveal excellent short-term biocompatibility and low cytotoxicity across various liver disease models. Cytotoxicity assessments via MTT or CCK-8 assays across multiple cell lines (HeLa, 4T1, HepG2, L02, and LX-2) consistently showed cell viability exceeding 80% at working concentrations. In vivo, intravenous administration of these probes did not induce significant body weight changes, organ damage (assessed by H&E staining), or hemolysis, confirming their good biocompatibility and low acute toxicity for living animal imaging applications (Table 3). Despite these positive acute toxicity profiles, the long-term pharmacokinetics of enzyme-responsive agents remain ambiguous. A primary concern is the in vivo metabolic clearance of the cleaved fluorophore backbones. Moreover, the risk of off-target activation by homologous enzymes in non-hepatic tissues could induce unintended systemic distribution, necessitating comprehensive chronic toxicity assessments prior to clinical trials.

#### 3.2.2. Ratiometric and Functional Probes for Oxidative Stress and Reactive Molecules

The physiological landscape of hepatic diseases is intricately interwoven with explosive imbalances in the local biochemical microenvironment, characterized by transient bursts of reactive oxygen species (ROS) and compensatory fluctuations in gasotransmitters. Because these reactive species possess extremely short half-lives and strong spatial diffusion tendencies, traditional single-channel probes are highly susceptible to concentration-dependent artifacts. To conquer this, Liu et al. introduced a series of ratiometric PA probes (PABDP1-4) for real-time tracking of the superoxide–glutathione (GSH) redox cycle [92]. Utilizing a catechol group, the PEG-modified probe PABDP4 undergoes reversible structural oxidation to benzoquinone upon encountering a superoxide burst, inducing a spectral redshift from 690 nm to 760 nm, which can be subsequently reduced back by endogenous GSH. Through dual-channel ratiometric (PA_760_/PA_690_) quantitative analysis, artifacts caused by light field gradients are eliminated.

To maximize the deep-tissue activation fold and overcome the narrow redshift spans that typically plague conventional ROS probes, Liu et al. engineered hemicyanine dyes into a super-ratiometric probe, AIH-OTF [93]. Strategically replacing the indole ring with a 7-azaindole heterocycle drastically suppressed fluorescence and redirected absorbed energy into thermoelastic expansion, massively amplifying the PA signal by 9.7 times. Triggered by specific nucleophilic cleavage under oxidative stress, this probe exhibited an unprecedented spectral redshift of up to 203 nm. In hyper-acute clinical scenarios such as ALF, nanomaterials provide complementary advantages. Wu et al. constructed a nanozyme-augmented PA nanoprobe that leverages localized ROS to catalyze the in situ generation of microbubbles [94]. The non-linear acoustic scattering and thermoelastic expansion of these nanocatalytic bubbles exponentially amplify the PA signal in deep liver tissues. This multicomponent decoding strategy was further extended with a bidirectional ratiometric nanoprobe (1-PAIN) targeting the hydroxyl radical (•OH) –H_2_S redox cycle [95], while Chen et al. utilized dinitrophenyl ether cleavage (CDR) to map dynamic cysteine depletion during early DILI [96].

Gasotransmitters serve as critical intracellular messengers, but are difficult to capture due to their volatility. Tan et al. developed a carbon monoxide (CO) -activated chemical structure transformation probe, RP, establishing a quantitative correlation between CO concentration fluctuations and linear PA signal drift, enabling self-calibrated measurement [97]. Fan et al. extended this paradigm to reactive nitrogen species with the Nitroxyl (HNO) -responsive ratiometric probe NF, which undergoes irreversible tautomerization to produce a dual-channel PA interconversion between 795 and 680 nm [98]. Pursuing higher fidelity, Liu et al. constructed a dual-modality, dual-ratiometric platform (DOP-CO) in which CO-triggered structural transformation causes simultaneous proportional shifts in both fluorescence and PA spectra, effectively canceling systemic errors [99]. The diagnostic potential of gasotransmitter-responsive probes has also reached hepatic oncology. Targeting the H_2_S -enriched microenvironment of colorectal cancer liver metastasis, Xu et al. developed the ratiometric probe Cy-HCy-H_2_S [100]. Localized H_2_S induces an intramolecular torsion and conjugated system cleavage, yielding a strong ratiometric enhancement (PA_720_/PA_760_) that facilitates the noninvasive localization of occult deep micro-metastases and navigates precise surgical excisions.

Beyond structural design, the in vivo safety of redox- and gas-responsive probes is another critical dimension. Preliminary toxicity assays across various molecular and nano-augmented platforms have generally shown favorable cellular tolerability. In vitro cytotoxicity assays (MTT or CCK-8) across multiple cell lines (e.g., HepG2, L02, RAW264.7, HeLa, HCT116, L929) consistently showed cell viability > 80–90% at working concentrations. In vivo assessments, including H&E staining of major organs, body weight monitoring, and serum biochemistry, confirmed negligible acute toxicity and good biocompatibility for those probes that were evaluated systemically (Table 4). The transition of these highly reactive probes into clinical settings is primarily hindered by the unpredictable biological fate of their reaction products. For instance, the long-term immunogenicity and potential hepatic retention of auxiliary nanocarriers (such as ceria nanozymes in RSPN) lack thorough investigation. Furthermore, it is imperative to prove that the oxidized or tautomerized residues of these probes will not provoke secondary oxidative stress during hepatobiliary or renal clearance.

#### 3.2.3. Probes Responding to Microenvironmental Physical Properties and Metal Ion Homeostasis

Hepatic microenvironment remodeling involves not only biochemical fluctuations but also profound shifts in biophysical properties and transition metal homeostasis, which have traditionally been assessed by invasive needle biopsy. During the progression of NAFLD and drug-induced fatty liver disease, massive intracellular lipid accumulation causes a sharp increase in intracellular viscosity. To noninvasively map this biophysical alteration, Zhang et al. designed an orally administrable dual-modality probe, WSP-3, based on the twisted intramolecular charge transfer (TICT) mechanism [101]. In healthy, low-viscosity environments, unrestricted intramolecular single-bond rotation dissipates absorbed energy via non-radiative thermal decay, optically silencing the PA signal. Conversely, in the highly viscous lipid microenvironment of fatty liver lesions, steric hindrance restricts this rotation, blocking the TICT process and turning on strong PA radiation. Crucially, this study innovated the delivery strategy by utilizing oral administration to fully exploit the liver’s “first-pass effect,” achieving high probe accumulation via the portal vein while minimizing non-specific systemic distribution.

Parallel to these biophysical changes, disrupted transition metal homeostasis represents another critical pathological axis. The pathological deposition of cuprous ions (Cu(I)) is a core feature of hereditary Wilson’s disease. To avoid traumatic biopsies, Lucero et al. introduced a “biopsy-free assessment” strategy by developing the activatable probe PACu-1 [102], which conjugates a highly selective Cu(I)-chelating pocket onto an aza-BODIPY platform. Upon metal embedding, efficient charge redistribution drives a robust, self-calibrating ratiometric spectral shift, allowing for the high-fidelity longitudinal monitoring of chronic metal metabolic disorders without quantitative errors from deep-tissue light gradients. The scope of noninvasive metal sensing has recently expanded to exogenous heavy metal contaminants. Addressing the toxicological threat of environmental palladium (Pd^2+^) exposure, Zhu et al. synthesized the first activatable near-infrared-II (NIR-II) fluorescent and ratiometric PA dual-modality probe, NYR-1 [103]. Utilizing the classic Tsuji–Trost allylic cleavage reaction as a specific detection valve for Pd^2+^ overload, the palladium-triggered cleavage perfectly restores the molecule’s ICT effect. This synchronously activates NIR-II fluorescence with ultralow tissue scattering and provides a powerful analytical tool for assessing early parenchymal detoxification impairment caused by environmental toxins.

Current in vivo evidence points to a high degree of safety during short-term monitoring windows for probes that respond to microenvironmental and metal ion fluctuations. In vitro cytotoxicity assays (CCK-8 or MTT) across various cell lines consistently showed high cell viability (>80%) at working concentrations. For WSP-3 and PACu-1, in vivo assessments including H&E staining of major organs and serum biochemistry (liver function tests) confirmed negligible acute toxicity. Additionally, WSP-3 exhibited no hemolytic reaction and good stability in serum and gastric acid, while PACu-1 showed metabolic stability in liver microsomes without off-target activation. NYR-1, though lacking extensive in vivo toxicity data, showed no significant cytotoxicity in cell studies and was successfully used for in vivo imaging without reported adverse effects (Table 5). The most distinct barrier for metal-chelating probes (e.g., PACu-1 and NYR-1) is the post-activation systemic toxicity. The thermodynamic stability of the probe-metal complexes must be strictly guaranteed during excretion. Premature dissociation can trigger heavy metal redistribution and severe nephrotoxicity. In a different vein, for viscosity sensors designed for oral administration (like WSP-3), mapping their long-term impact on the gastrointestinal microbiome is a mandatory prerequisite for future human studies.

**Figure 5:**
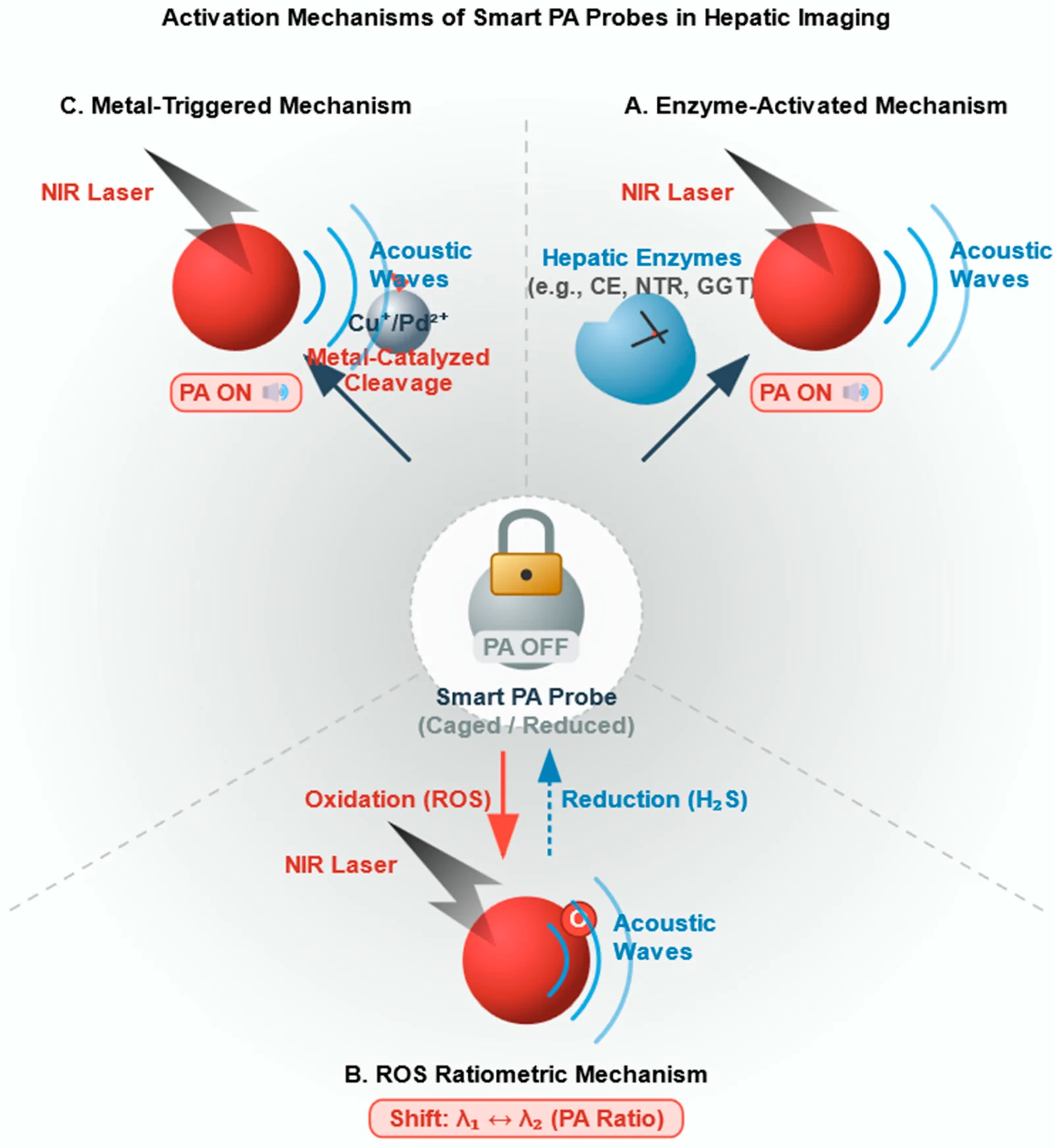
Comprehensive overview of activation mechanisms for smart photoacoustic (PA) probes in the hepatic microenvironment. The central core represents the generic smart PA probe in its initial caged or reduced state (PA OFF), where the intramolecular charge transfer (ICT) is blocked. From the center, three distinct pathological activation pathways are illustrated. (**A**) Enzyme-activated mechanism: overexpressed hepatic enzymes (e.g., CE, NTR, GGT) specifically cleave the recognition moiety, restoring the conjugated system and turning the PA signal “ON.” (**B**) ROS/gas-responsive ratiometric mechanism: oxidation by reactive oxygen species (ROS) induces a structural rearrangement, leading to a bathochromic shift from the wavelength (λ_1_) to a longer wavelength (λ_2_), enabling ratiometric PA imaging (PA_λ_2_/PA_λ_1_). This process can be reversibly reduced by endogenous gases like H_2_S. (**C**) Metal-triggered mechanism: in cases of hepatic metal overload (e.g., Wilson’s disease or environmental exposure), specific metal ions (Cu^+^, Pd^2+^) catalyze the cleavage of the masking ligand, releasing the active fluorophore and converting absorbed optical energy (NIR laser) into strong acoustic waves.

In conclusion, strategies for designing activatable photoacoustic probes for hepatic imaging have evolved into three primary categories based on different pathological triggers. Figure 5 summarizes and contrasts these distinct activation mechanisms, namely the enzyme-cleavable “OFF-to-ON” approach, the ROS-responsive ratiometric strategy, and the metal ion-catalyzed cleavage mechanism.

## 4. Discussion

Although histopathological biopsy and repeated invasive serological sampling (such as in vitro ICG clearance tests) remain the gold standards for diagnosing liver diseases and assessing LFR, these invasive methods carry significant drawbacks, including procedural risks, sampling errors, and the inability to achieve real-time dynamic monitoring. With the continuous development of novel tools and probes, photoacoustic imaging (PAI) has been increasingly applied for rapid, efficient, panoramic, and precise assessment of conditions ranging from acute drug-induced liver injury (DILI) to chronic MASLD and liver fibrosis.

PAI offers multidimensional advantages in the diagnosis of liver diseases over traditional medical imaging. First, portable handheld PAI systems have enabled noninvasive bedside monitoring of endogenous chromophore metabolism and exogenous ICG clearance kinetics [104], drastically shortening examination time and eliminating the pain associated with invasive procedures. Second, integrated with smart activatable probes, PAI overcomes the latency of traditional imaging, which typically only observes macroscopic anatomical alterations. It achieves precise molecular sensing of biochemical microenvironmental changes (e.g., specific enzyme fluctuations, ROS bursts, and heavy metal accumulation) at very early disease stages [105]. This ability to detect imminent macroscopic consequences from early microscopic signals is expected to facilitate the integration of PAI into future clinical diagnostic matrices for the early warning and intervention of chronic liver diseases.

However, the clinical translation of hepatic PAI remains challenged by both engineering and biochemical limitations. In humans, the abdominal wall, comprising skin, subcutaneous fat, and muscle, has a mean subcutaneous fat thickness of several centimeters in adults and can be substantially thicker in obese patients. The human liver itself extends to a total depth of about 8–10 cm. Although a pilot study achieved transcutaneous lipid imaging at a depth of 3.4 cm in patients with hepatic steatosis [70], this depth is sufficient only to reach the liver surface, not to image the full organ. Moreover, the 930 nm lipid signal dropped by about 83% between the subcutaneous layer (0.4 cm) and the first liver segment (2.5 cm) and by a further ~15% per centimeter thereafter, yielding an SNR of 4.97 at 3.4 cm, which underscores the steep attenuation that transcutaneous hepatic PAI must overcome. By contrast, in small-animal models, the abdominal wall is typically only about 1 mm thick and the liver can be imaged within a depth of 1–2 cm, whereas PAT can provide sufficient SNR at depths of more than 4 cm. Blindly increasing laser energy to probe deeper targets poses a risk of thermal injury, despite promising hardware innovations such as dynamic light delivery [106]. Laser exposure must follow clinically proven safety limits. Respiratory and cardiac motion, which can introduce 1–2 cm liver displacement in humans, while largely minimized in anesthetized and gated small-animal studies, affects spectral unmixing quality and quantitative reproducibility in human studies. Quantitative PAI is further limited by optical fluence attenuation and compensation, spectral unmixing errors, and acoustic heterogeneity. Because the PA signal depends on both absorption and local fluence, separating them is ill-posed: direct fluence measurement is invasive, and model-based corrections assume homogeneity that the human liver violates. Linear spectral unmixing with crude fluence correction is often inaccurate, as endogenous chromophores have overlapping spectra and wavelength-dependent fluence distorts apparent spectra. Reconstruction typically assumes a uniform sound speed (~1540 m/s), but lipid-rich and fibrotic regions have different speeds, causing phase aberration and quantitative artifacts. Acoustic coupling also requires careful adaptation. Conventional coupling gels absorb near-infrared light, limiting penetration. Although heavy water (D_2_O) can reduce this absorption, its use as an acoustic coupling medium requires adaptation to clinical probe geometries. Finally, handheld probe operation introduces operator dependence, and data analysis often relies on experienced clinicians, while the scan time required for multispectral acquisition constrains throughput. Biochemically, the actual long-term biological toxicity, immunogenicity, and in vivo metabolic pathways of most ratiometric smart probes remain unknown, hindering regulatory approval by agencies such as the FDA. Other key barriers include uncertainties in pharmacokinetics and biodistribution, insufficient targeting specificity in the complex human hepatic environment, challenges in achieving reproducible large-scale synthesis, and the lack of standardized dosing and regulatory pathways for optoacoustic contrast agents. Thus, most probes remain at the stage of proof-of-concept validation in small-animal models, and systematic evaluation in large animals or humans is still largely absent. Consequently, hepatic PAI is still largely confined to preclinical studies using simplified animal models (e.g., APAP-induced DILI) that do not fully reflect the highly complex, multifactorial, and slowly progressive nature of human liver diseases.

To overcome these translational barriers, future efforts are rapidly converging on several key directions. Miniaturization toward point-of-care (POC) architectures is critical. Compact handheld systems will facilitate flexible bedside monitoring in intensive care units and real-time intraoperative navigation. Photoacoustic endoscopy has already achieved clinical pilot results in the gastrointestinal tract [107], and these technological advances, including miniaturized ultrasound transducers, fiber-based illumination, spectral unmixing, and AI-assisted interpretation, can be directly transferred to endoscopic photoacoustic probes for liver applications [108]. Internal light illumination in photoacoustic endoscopy, delivered via optical fibers inserted into body cavities or through interventional access, bypasses the optical attenuation of the abdominal wall and has been shown to extend photoacoustic imaging depth to 60 to 100 mm from the skin surface or beyond 10 cm in optimized configurations [109]. Concurrently, cost reduction is essential for clinical adoption. The high cost and intense maintenance demands of traditional Nd:YAG pumped optical parametric oscillator (OPO) lasers currently limit accessibility. Shifting toward affordable, robust solid-state sources such as laser diodes (LDs) and light-emitting diodes (LEDs) [110], will substantially lower financial barriers. Furthermore, advancing computational methods toward absolute quantification will define the next generation of hepatic PAI. Moving beyond qualitative contrast enhancement, integrating deep-learning and advanced computational models [40] can dynamically compensate for localized light fluence variations and acoustic heterogeneities, thereby yielding highly reproducible, operator-independent quantitative biomarkers. Multimodal integration represents a major diagnostic trend [111]. For hepatology, a seamlessly integrated photo trimodal acoustic, ultrasound, and elastography system could simultaneously assess liver stiffness for fibrosis staging, provide anatomical landmarks via B-mode ultrasound, and deliver molecular metabolic profiling [55]. Such a synergistic platform may be capable of rivaling current clinical gold standards. Future multimodal systems may further integrate PAI with ultrafast ultrasound to provide a more comprehensive assessment of hepatic perfusion and vascular remodeling.

## 5. Conclusions

In conclusion, driven by the steadily escalating global burden of acute and chronic liver diseases and the urgent clinical demand for early, noninvasive, and precise diagnosis, this review has systematically summarized recent advances in photoacoustic hepatic imaging systems and molecular probes. These technologies are now widely applied in intravital studies of complex liver disease models and early clinical explorations. A powerful diagnostic tool, PAI uniquely integrates deep-penetration, whole-liver quantitative evaluation with microscopic functional and structural assessment, both of which correlate strongly with the current gold standards of histopathological biopsy and serological testing. Given the multifaceted nature of liver disease, no single noninvasive imaging modality can fully capture the structural, hemodynamic, and molecular alterations required for comprehensive diagnosis. Multimodal integration therefore represents the most promising path forward, and PAI is a naturally fit. By combining optical contrast with deep ultrasound penetration, PAI can be seamlessly merged with established clinical tools such as ultrasound and fluorescence imaging to form a unified, multiparametric diagnostic platform. Realizing this vision, however, demands substantial reductions in system cost and footprint, moving from bulky, expensive laser sources toward compact, affordable solid-state alternatives. Equally important, the rapid evolution of artificial intelligence (AI) will profoundly accelerate the clinical translation of PAI, enabling sparse sampling, real-time fluence compensation, and the extraction of reproducible quantitative biomarkers from complex multimodal datasets. With continued advances in probe biosafety and deep-tissue light delivery, such a platform could ultimately deliver a comprehensive, cross-scale portrait of hepatic pathology and benefit patients worldwide.

## Figures and Tables

**Table 1:** Comparison of representative PACT platforms and technologies.

System/Technology	SRCPT [43]	3D-PAT [47]	DL-PACT [49]	3D-multiparametric PACT [46]	HD-PACT [50]
Level	Method	Generic representative implementation	System	System	System
Detection Platform	512-element full-ring array, 5.5 MHz, ring diameter 100 mm	4 × 256-element arc arrays, 2.25 MHz, >98% one-way bandwidth	1024-element hemispherical array, 2.02 MHz, 54% bandwidth	same as DL-PACT platform	same as DL-PACT platform
Reconstruction/Enhancement Algorithm	FCS-SAFT (frequency component selection synthetic aperture focusing) + MC-EMM (Monte Carlo-corrected empiric mathematic model) fluence compensation	Dual-speed UBP + respiratory gating motion correction	3D progressive U-net (cluster view with 1/4 elements can reach full-array quality)	Custom delay-and-sum (CUDA accelerated)	Hybrid diffusion model (256 sparse to 1024 full-array paired training; minimum 128 elements)
Spatial Resolution	lateral 0.12 mm; elevational 0.6 mm	~380 µm	~380 µm (full array); cluster view XY ~700 µm, Z ~300 µm	~380 µm (isotropic)	~380 µm (full-array)
FOV	NR^1^	16 cm (larger than average adult liver)	12.8 × 12.8 × 12.8 mm	12.8 × 12.8 × 12.8 mm (scan-free); whole mouse body (raster stitching)	12.8 × 12.8 × 12.8 mm
Imaging Speed	20 Hz (laser repetition rate)	50 Hz (Nd:YAG laser repetition rate); 3D volume frame rate not specified	Full view 5 Hz; cluster view 20 Hz; DL real-time up to 62.5 Hz	20 Hz (laser repetition rate); whole-body acquisition < 30 s	5 Hz (1024 full array)
Penetration/Imaging Depth	NR^1^ (not given; deep liver/kidney clearance imaging achieved)	1.2 cm (rat liver imaging depth); 730 nm expected 5 cm depth with SNR 3× of 1064 nm (original)	NR^1^ (not given; deep brain/torso/kidney imaging in rats)	~9 mm (756 nm whole-body imaging)	NR^1^ (not given; rat + human hand imaging)
Wavelength	1064 nm (structural and tracking); 750/850/780/774 nm for functional and tracking	1064 nm	690–1064 nm (OPO, 20 Hz); DL training at 900 nm	730/756/796/866 nm (sO_2_); 780 nm (pharmacokinetics)	Training 900 nm; functional imaging 730/756/796/866 nm; generalization validation also includes 800 nm
Representative Validation	Dynamic tracking of liver/kidney drug clearance pathways, strong positive correlation with dual-labeled PET in mice	Lean/obese rat liver angiography and NAFLD assessment	Brain sO_2_/oxy- (HbO) and deoxy-hemoglobin (HbR) dynamics, pharmacokinetic tracking in mice	Primary/metastatic liver tumor angiogenesis, hypoxia, pharmacokinetics in mice	Cross-plane (mice), cross-organism (mice to human palm), cross-wavelength generalization; sO_2_/HbT

^1^ All measurable data presented in this table were extracted from the cited references. NR, not reported: the corresponding parameter was not documented in the source literature and no indirect inference was performed.

**Table 2:** Technical specifications of representative commercial PAT systems.

System (Manufacturer)	SIP-PACT [64] (Union Photoacoustic Technologies)	LOIS-3D [65] (TomoWave Laboratories)	MSOT inVision 256-TF [60] (iThera Medical)	Vevo LAZR [66] (FUJIFILM VisualSonics)
Spatial Resolution	125 μm in-plane [64]	150 μm, 3D isotropic [67]	150 μm in-plane [68]	down to 45 μm [66]
2D Frame Rate	50 Hz [64]	NR^1^	10 Hz [68]	5–20 Hz [66]
Field of View (FOV)	up to 48 mm in width	≥40 × 40 × 40 mm/pulse [67]	≥25 mm [69]	14–23 mm wide, 10 mm long [66]
Penetration Depth	rat whole brain (~10 mm in depth) [64]	>4.5 cm in mouse [67]	30–40 mm [70]	10 mm [66]
Optical Absorption Sensitivity	NR^1^	0.03 cm^−1^ (~1 pM ICG) [67]	50 nM ICG [68]	521 nM Methylene Blue [66]
Excitation Geometry	top conical/full-ring [64]	2 orthogonal, 2 oblique lasers [67]	360° full ring [60]	1 mm × 24 mm line [66]
Wavelength Range	1064 nm, 630 nm, 680 nm, 720 nm [64]	660–1064 nm/660–2300 nm [67]	680–980 nm [68]	680–970 nm [66]
Single-Pulse Energy	>1 J [71]	≥180 mJ [67]	≤100 mJ [68]	45 mJ ± 5 mJ (at 20 Hz) [66]
Pulse Duration	5–9 ns @ 1064 nm, 12 ns @ 720 nm [64]	6 ns [72]	9 ns [73]	4–6 ns [66]
Laser Exposure	<100 mJ/cm^2^ [71]	NR^1^	NR^1^	<20 mJ/cm^2^ [74]
Detection Geometry	full ring, 512 elements [64]	120° arc-shaped [67]	270° arc-shaped, 256 elements [60]	linear, 256 elements [66]
Transducer Frequency	5 MHz center, >90% one-way bandwidth [64]	0.05–8 MHz [72]	5 MHz center, >55% bandwidth [68]	32 MHz–55 MHz (LZ550 transducer)/13 MHz–24 MHz (LZ250 transducer) [66]

^1^ NR, not reported: the corresponding parameter was not provided in the cited references. All measurable data presented in this table were extracted from the cited references. These values were obtained under different experimental conditions and should therefore be interpreted with caution.

**Table 3:** Summary of enzyme-activatable photoacoustic probes.

Probe Name	Response Mechanism	Signal Change/Spectral Shift	Liver Disease Model	Toxicity/Biosafety
QHD-CE	Specific ester bond cleavage	Signal gradient decrease (ON → OFF)	Drug-induced liver injury (DILI)	Low cytotoxicity (~80% HepG2 viability, 24 h); no obvious hepatotoxicity in vivo up to 24 h
NO_2_-CS	Cascade reduction of nitro to amino group	Relief of optical silence (OFF → ON)	Isoniazid-induced early hepatic hypoxia	Cell viability > 80% at 30 μM (24 h); excellent biocompatibility
HDP-GGT	Enzymatic activation and cleavage	NIR absorption turn-on (OFF → ON)	Diabetic mouse model (Hepatoprotective evaluation)	No significant effect on body weight over 4 days; negligible in vivo toxicity
ETYZE-GGT	Enzymatic activation and cleavage	NIR absorption turn-on (OFF → ON)	Autoimmune hepatitis/Early hepatocellular carcinoma	>90% cell survival at 50 μM; no adverse effects after 7-day repeated IV (3.5 mg/kg)
IP	Receptor anchoring + Enzymatic activation	Targeted PA signal turn-on (OFF → ON)	Liver fibrosis (LF)	>85% viability in L02 cells; negligible hemolysis up to 200 μM; no organ damage at 24 h

**Table 4:** Summary of ratiometric and functional photoacoustic probes for oxidative stress and reactive molecules in the liver.

Target/Biomarker	Probe Name	Response Mechanism	Signal Change/Spectral Shift	Liver Disease Model	Toxicity/Biosafety
Superoxide anion (O_2_•^−^)/GSH	PABDP4	Catechol oxidation/benzoquinone reduction	Ratiometric reversible shift (690/760 nm)	APAP-induced acute liver injury	Not reported
O_2_•^−^	AIH-OTF	Nucleophilic cleavage relieving ICT inhibition	Massive redshift (203 nm) and 9.7-fold enhancement	APAP-induced acute liver injury	No significant cytotoxicity up to 50 μM in HepG2 cells; no abnormal lesions in major organs
ROS	RSPN	Nanoenzyme-catalyzed gas generation	Amplification of thermoelastic expansion	Severe acute liver failure	Excellent in vivo biocompatibility; no significant changes in serum biochemistry or routine blood parameters
•OH/H_2_S	1-PAIN	Bidirectional redox reversible response	Bidirectional ratiometric shift (PA_690_/PA_825_)	Early imbalance in liver inflammation	Good biocompatibility in RAW264.7 macrophages; no obvious inflammatory response or systemic toxicity after IV
Cysteine	CDR	Specific cleavage of dinitrophenyl ether	Synchronous turn-on of dual-modality signals	Drug-induced liver injury	>80% viability at 50 μM; no casualties and normal body weight gain in mice
CO	RP	Specific chemical structural transformation valve	Linear shift response in absorption spectrum	DILI and its detoxification/repair process	>90% viability in HeLa cells at 50 μM
HNO	NF	Response-induced structural interconversion	Dual-channel ratiometric shift (795/680 nm)	Prodrug release and hepatic metabolic injury	Low cytotoxicity in L02 cells
CO	DOP-CO	Specific chemical structural transformation	Dual-modality and dual-ratiometric self-calibration	Precise quantification of APAP-induced DILI	Negligible cytotoxicity; favorable for biological applications
H_2_S	Cy-HCy-H_2_S	Cyanine-to-hemicyanine transformation	Ratiometric self-calibrating shift (760 to 720 nm)	Colorectal cancer (CRC) liver metastasis targeting	>85% viability in HCT116 and L929 cells at 20 μM for 24 h

**Table 5:** Summary of photoacoustic probes responding to microenvironmental physical properties and metal ion homeostasis.

Target/Biomarker	Probe Name	Response Mechanism	Signal Change/Spectral Shift	Liver Disease Model	Toxicity/Biosafety
Microenvironmental viscosity	WSP-3	Twisted intramolecular charge transfer (TICT)	Oral targeting, turn-on via restricted rotation	Drug-induced/Non-alcoholic fatty liver disease	>80% viability in HeLa and HepG2 cells up to 40 μM; no hemolysis; no significant organ lesions after oral 400 μM
Cuprous ion (Cu^+^)	PACu-1	Ion chelation-triggered backbone rearrangement	Biopsy-free ratiometric shift self-calibration	Wilson’s disease/Metastatic tumor angiogenesis	High viability in HEK293 cells up to 25 μM; non-cytotoxic product; liver H&E and function tests comparable to controls
Palladium ion (Pd^2+^)	NYR-1	Tsuji-Trost allylic cleavage	NIR-II dual-channel ratiometric interconversion	Overload exposure to heavy metal environmental toxin	No significant cytotoxicity in HeLa cells within tested dose range

## Data Availability

No new data were created or analyzed in this study. Data sharing is not applicable to this article.

## References

[1] Devarbhavi H., Asrani S.K., Arab J.P., Nartey Y.A., Pose E., Kamath P.S. (2023). Global Burden of Liver Disease: 2023 Update. J. Hepatol..

[2] Naghavi M., Kyu H.H., A B., Aalipour M.A., Aalruz H., Ababneh H.S., Abafita B.J., Abaraogu U.O., Abbafati C., Abbasi M. (2025). Global Burden of 292 Causes of Death in 204 Countries and Territories and 660 Subnational Locations, 1990–2023: A Systematic Analysis for the Global Burden of Disease Study 2023. Lancet.

[3] World Health Organization (2026). Global Hepatitis Report 2026.

[4] Sung H., Filho A.M., Laversanne M., Ferlay J., Siegel R.L., Soerjomataram I., Jemal A., Bray F. (2026). Global Cancer Statistics 2024: GLOBOCAN Estimates of Incidence and Mortality Worldwide for 34 Cancers in 186 Countries. CA Cancer J. Clin..

[5] Karvellas C.J., Leventhal T.M., Rakela J.L., Zhang J., Durkalski V., Reddy K.R., Fontana R.J., Stravitz R.T., Lake J.R., Lee W.M. (2023). Outcomes of Patients with Acute Liver Failure Listed for Liver Transplantation: A Multicenter Prospective Cohort Analysis. Liver Transplant..

[6] Zhao Y., Bo Y., Zu J., Xing Z., Yang Z., Zhang Y., Deng Y., Liu Y., Zhang L., Yuan X. (2025). Global Burden of Chronic Liver Disease and Temporal Trends: A Population-Based Analysis From 1990 to 2021 With Projections to 2050. Liver Int..

[7] Younossi Z.M., Kalligeros M., Henry L. (2025). Epidemiology of Metabolic Dysfunction-Associated Steatotic Liver Disease. Clin. Mol. Hepatol..

[8] Ho G.J.K., Tan F.X.N., Sasikumar N.A., Tham E.K.J., Ko D., Kim D.H., Danpanichkul P., Yu Z., Xianda C., Zhang Z.X. (2025). High Global Prevalence of Steatotic Liver Disease and Associated Subtypes: A Meta-Analysis. Clin. Gastroenterol. Hepatol..

[9] McSteen B.W., Ying X.-H., Lucero C., Jesudian A.B. (2024). Viral Etiologies of Acute Liver Failure. World J. Virol..

[10] Maiwall R., Kulkarni A.V., Arab J.P., Piano S. (2024). Acute Liver Failure. Lancet.

[11] Shalimar, Acharya S.K., Kumar R., Bharath G., Rout G., Gunjan D., Nayak B. (2020). Acute Liver Failure of Non–A-E Viral Hepatitis Etiology—Profile, Prognosis, and Predictors of Outcome. J. Clin. Exp. Hepatol..

[12] Moon A.M., Singal A.G., Tapper E.B. (2020). Contemporary Epidemiology of Chronic Liver Disease and Cirrhosis. Clin. Gastroenterol. Hepatol..

[13] Gan C., Yuan Y., Shen H., Gao J., Kong X., Che Z., Guo Y., Wang H., Dong E., Xiao J. (2025). Liver Diseases: Epidemiology, Causes, Trends and Predictions. Signal Transduct. Target. Ther..

[14] Berzigotti A., Tsochatzis E., Boursier J., Castera L., Cazzagon N., Friedrich-Rust M., Petta S., Thiele M. (2021). EASL Clinical Practice Guidelines on Non-Invasive Tests for Evaluation of Liver Disease Severity and Prognosis–2021 Update. J. Hepatol..

[15] Castera L., Friedrich-Rust M., Loomba R. (2019). Noninvasive Assessment of Liver Disease in Patients With Nonalcoholic Fatty Liver Disease. Gastroenterology.

[16] Catania R., Furlan A., Smith A.D., Behari J., Tublin M.E., Borhani A.A. (2021). Diagnostic Value of MRI-Derived Liver Surface Nodularity Score for the Non-Invasive Quantification of Hepatic Fibrosis in Non-Alcoholic Fatty Liver Disease. Eur. Radiol..

[17] Chou R., Cuevas C., Fu R., Devine B., Wasson N., Ginsburg A., Zakher B., Pappas M., Graham E., Sullivan S.D. (2015). Imaging Techniques for the Diagnosis of Hepatocellular Carcinoma. Ann. Intern. Med..

[18] Beard P. (2011). Biomedical Photoacoustic Imaging. Interface Focus..

[19] Wang L.V., Hu S. (2012). Photoacoustic Tomography: In Vivo Imaging from Organelles to Organs. Science.

[20] Xia J., Yao J., Wang L.V. (2014). Photoacoustic Tomography: Principles and Advances (Invited Review). Prog. Electromagn. Res..

[21] Wang L.V. (2009). Multiscale Photoacoustic Microscopy and Computed Tomography. Nat. Photonics..

[22] Yao J., Wang L.V. (2013). Photoacoustic Microscopy. Laser Photon. Rev..

[23] Zhang H.F., Maslov K., Stoica G., Wang L. (2006). V Functional Photoacoustic Microscopy for High-Resolution and Noninvasive In Vivo Imaging. Nat. Biotechnol..

[24] Maslov K., Zhang H.F., Hu S., Wang L.V. (2008). Optical-Resolution Photoacoustic Microscopy for In Vivo Imaging of Single Capillaries. Opt. Lett..

[25] Li C., Wang L. (2009). V Photoacoustic Tomography and Sensing in Biomedicine. Phys. Med. Biol..

[26] Gu Y., Sun Y., Wang X., Li H., Qiu J., Lu W. (2023). Application of Photoacoustic Computed Tomography in Biomedical Imaging: A Literature Review. Bioeng. Transl. Med..

[27] Hu S., Wang L.V. (2010). Photoacoustic Imaging and Characterization of the Microvasculature. J. Biomed. Opt..

[28] Maslov K., Stoica G., Wang L.V. (2005). In Vivo Dark-Field Reflection-Mode Photoacoustic Microscopy. Opt. Lett..

[29] Kruger R.A., Liu P., Fang Y., Appledorn C.R. (1995). Photoacoustic Ultrasound (PAUS)—Reconstruction Tomography. Med. Phys..

[30] Menozzi L., Yao J. (2024). Deep Tissue Photoacoustic Imaging with Light and Sound. npj Imaging.

[31] Kim W., Choi W., Ahn J., Lee C., Kim C. (2023). Wide-Field Three-Dimensional Photoacoustic/Ultrasound Scanner Using a Two-Dimensional Matrix Transducer Array. Opt. Lett..

[32] Lin L., Hu P., Tong X., Na S., Cao R., Yuan X., Garrett D.C., Shi J., Maslov K., Wang L.V. (2021). High-Speed Three-Dimensional Photoacoustic Computed Tomography for Preclinical Research and Clinical Translation. Nat. Commun..

[33] Yang J., Choi S., Kim C. (2022). Practical Review on Photoacoustic Computed Tomography Using Curved Ultrasound Array Transducer. Biomed. Eng. Lett..

[34] Zhang Y., Wang L. (2022). Video-Rate Full-Ring Ultrasound and Photoacoustic Computed Tomography with Real-Time Sound Speed Optimization. Biomed. Opt. Express..

[35] Li Y., Li L., Zhu L., Maslov K., Shi J., Hu P., Bo E., Yao J., Liang J., Wang L. (2020). Snapshot Photoacoustic Topography through an Ergodic Relay for High-Throughput Imaging of Optical Absorption. Nat. Photonics..

[36] Wang X., Meng Y., Sun M., Gao X., Wang Y., Wang S., Wang K., Wang R., Ren D., Yin Y. (2025). Cross-Regional Real-Time Visualization of Systemic Physiology and Dynamics with 3D Panoramic Photoacoustic Computed Tomography (3D-PanoPACT). Nat. Commun..

[37] Rosenthal A., Ntziachristos V., Razansky D. (2014). Acoustic Inversion in Optoacoustic Tomography: A Review. Curr. Med. Imaging Rev..

[38] Xu M., Wang L.V. (2005). Universal Back-Projection Algorithm for Photoacoustic Computed Tomography. Phys. Rev. E.

[39] Deng H., Qiao H., Dai Q., Ma C. (2021). Deep Learning in Photoacoustic Imaging: A Review. J. Biomed. Opt..

[40] Yang C., Lan H., Gao F., Gao F. (2021). Review of Deep Learning for Photoacoustic Imaging. Photoacoustics.

[41] Zhang F., Zhang J., Shen Y., Gao Z., Yang C., Liang M., Gao F., Liu L., Zhao H., Gao F. (2023). Photoacoustic Digital Brain and Deep-Learning-Assisted Image Reconstruction. Photoacoustics.

[42] Hauptmann A., Lucka F., Betcke M., Huynh N., Adler J., Cox B., Beard P., Ourselin S., Arridge S. (2018). Model-Based Learning for Accelerated, Limited-View 3-D Photoacoustic Tomography. IEEE Trans. Med. Imaging..

[43] Lv J., Lan H., Qin A., Sun T., Shao D., Gao F., Yao J., Avanaki K., Nie L. (2024). Dynamic Synthetic-Scanning Photoacoustic Tracking Monitors Hepatic and Renal Clearance Pathway of Exogeneous Probes In Vivo. Light Sci. Appl..

[44] Deán-Ben X.L., Fehm T.F., Ford S.J., Gottschalk S., Razansky D. (2016). Spiral Volumetric Optoacoustic Tomography Visualizes Multi-Scale Dynamics in Mice. Light Sci. Appl..

[45] Gottschalk S., Degtyaruk O., Mc Larney B., Rebling J., Hutter M.A., Deán-Ben X.L., Shoham S., Razansky D. (2019). Rapid Volumetric Optoacoustic Imaging of Neural Dynamics across the Mouse Brain. Nat. Biomed. Eng..

[46] Kim J., Lee J., Choi S., Lee H., Yang J., Jeon H., Sung M., Kim W.J., Kim C. (2024). 3D Multiparametric Photoacoustic Computed Tomography of Primary and Metastatic Tumors in Living Mice. ACS Nano.

[47] Tong X., Lin L., Hu P., Cao R., Zhang Y., Olick-Gibson J., Wang L.V. (2023). Non-Invasive 3D Photoacoustic Tomography of Angiographic Anatomy and Hemodynamics of Fatty Livers in Rats. Adv. Sci..

[48] Wang X., Xiao Y., Wang Y., Gao X., Ruan Y., Yang X., Su Y., Zhou D., Li X., Meng Y. (2026). Three-Dimensional Aperture-Driven Photoacoustic Tomography (3D-ADPAT). Sci. Adv..

[49] Choi S., Yang J., Lee S.Y., Kim J., Lee J., Kim W.J., Lee S., Kim C. (2023). Deep Learning Enhances Multiparametric Dynamic Volumetric Photoacoustic Computed Tomography In Vivo (DL-PACT). Adv. Sci..

[50] Jeong H., Oh S., Choi S., Kim J., Yang J., Kim C. (2026). A Hybrid Diffusion Model Enhances Multiparametric 3D Photoacoustic Computed Tomography. Adv. Sci..

[51] Krull A., Buchholz T.-O., Jug F. (2019). Noise2Void–Learning Denoising From Single Noisy Images. Proceedings of the 2019 IEEE/CVF Conference on Computer Vision and Pattern Recognition (CVPR).

[52] Lehtinen J., Munkberg J., Hasselgren J., Laine S., Karras T., Aittala M., Aila T. Noise2Noise: Learning Image Restoration without Clean Data. Proceedings of the Proceedings of the 35th International Conference on Machine Learning.

[53] Mansour Y., Heckel R. (2023). Zero-Shot Noise2Noise: Efficient Image Denoising without Any Data. Proceedings of the 2023 IEEE/CVF Conference on Computer Vision and Pattern Recognition (CVPR).

[54] Awasthi N., Jain G., Kalva S.K., Pramanik M., Yalavarthy P.K. (2020). Deep Neural Network-Based Sinogram Super-Resolution and Bandwidth Enhancement for Limited-Data Photoacoustic Tomography. IEEE Trans. Ultrason. Ferroelectr. Freq. Control..

[55] Lafci B., Hadjihambi A., Determann M., Konstantinou C., Freijo C., Herraiz J.L., Blümel S., Pellerin L., Burton N.C., Deán-Ben X.L. (2023). Multimodal Assessment of Non-Alcoholic Fatty Liver Disease with Transmission-Reflection Optoacoustic Ultrasound. Theranostics.

[56] Godefroy G., Arnal B., Bossy E. (2023). Full-Visibility 3D Imaging of Oxygenation and Blood Flow by Simultaneous Multispectral Photoacoustic Fluctuation Imaging (MS-PAFI) and Ultrasound Doppler. Sci. Rep..

[57] Zhao S., Hartanto J., Joseph R., Wu C.-H., Zhao Y., Chen Y.-S. (2023). Hybrid Photoacoustic and Fast Super-Resolution Ultrasound Imaging. Nat. Commun..

[58] Chen H., Mirg S., Gaddale P., Agrawal S., Li M., Nguyen V., Xu T., Li Q., Liu J., Tu W. (2024). Multiparametric Brain Hemodynamics Imaging Using a Combined Ultrafast Ultrasound and Photoacoustic System. Adv. Sci..

[59] Huang S., Blutke A., Feuchtinger A., Klemm U., Zachariah Tom R., Hofmann S.M., Stiel A.C., Ntziachristos V. (2021). Functional Multispectral Optoacoustic Tomography Imaging of Hepatic Steatosis Development in Mice. EMBO Mol. Med..

[60] Dima A., Burton N.C., Ntziachristos V. (2014). Multispectral Optoacoustic Tomography at 64, 128, and 256 Channels. J. Biomed. Opt..

[61] Huang G., Lv J., He Y., Yang J., Zeng L., Nie L. (2022). In Vivo Quantitative Photoacoustic Evaluation of the Liver and Kidney Pathology in Tyrosinemia. Photoacoustics.

[62] Needles A., Heinmiller A., Sun J., Theodoropoulos C., Bates D., Hirson D., Yin M., Foster F.S. (2013). Development and Initial Application of a Fully Integrated Photoacoustic Micro-Ultrasound System. IEEE Trans. Ultrason. Ferroelectr. Freq. Control..

[63] Ermilov S.A., Su R., Conjusteau A., Anis F., Nadvoretskiy V., Anastasio M.A., Oraevsky A.A. (2016). Three-Dimensional Optoacoustic and Laser-Induced Ultrasound Tomography System for Preclinical Research in Mice: Design and Phantom Validation. Ultrason. Imaging..

[64] Li L., Zhu L., Ma C., Lin L., Yao J., Wang L., Maslov K., Zhang R., Chen W., Shi J. (2017). Single-Impulse Panoramic Photoacoustic Computed Tomography of Small-Animal Whole-Body Dynamics at High Spatiotemporal Resolution. Nat. Biomed. Eng..

[65] Brecht H.-P.F., Su R., Fronheiser M.P., Ermilov S.A., Conjusteau A., Oraevsky A.A. (2009). Whole-Body Three-Dimensional Optoacoustic Tomography System for Small Animals. J. Biomed. Opt..

[66] Vevo® LAZR System Tech Specs. https://www.visualsonics.com/sites/default/files/Vevo_LAZR_technical_specifications.pdf.

[67] TomoWave Suzhou Medical Imaging Co., Ltd.. http://tomowave.net/LOIS3D_en.html.

[68] iThera Medical Optoakustický Tomografický Scanner MSOT inVision 256-TF Leaflet ENG.pdf. https://27b0c15159.clvaw-cdnwnd.com/ceb37299f62a5115396634193b95a22c/200002177-be4cfbf463/iThera%20Medical%20Optoakustick%C3%BD%20tomografick%C3%BD%20scanner%20MSOT%20inVision%20256-TF%20leaflet%20ENG.pdf.

[69] Muñiz-García A., Pichardo A.H., Littlewood J., Sharkey J., Wilm B., Peace H., O’Callaghan D., Green M., Taylor A., Murray P. (2023). Near Infrared Conjugated Polymer Nanoparticles (CPN^TM^) for Tracking Cells Using Fluorescence and Optoacoustic Imaging. Nanoscale Adv..

[70] Fasoula N.A., Karlas A., Prokopchuk O., Katsouli N., Bariotakis M., Liapis E., Goetz A., Kallmayer M., Reber J., Novotny A. (2023). Non-Invasive Multispectral Optoacoustic Tomography Resolves Intrahepatic Lipids in Patients with Hepatic Steatosis. Photoacoustics.

[71] CalPACT/Union Photoacoustic Technologies. http://www.union-pa.com/en/col.jsp?id=101.

[72] LOIS-3D Laser Optoacoustic Imaging System Brochure 2015. https://www.tomowave.com/wp-content/uploads/finalbrochure_lois-3d_2015.pdf.

[73] Morscher S., Driessen W.H.P., Claussen J., Burton N.C. (2014). Semi-Quantitative Multispectral Optoacoustic Tomography (MSOT) for Volumetric PK Imaging of Gastric Emptying. Photoacoustics.

[74] Vevo LAZR Application Note: Imaging of Vasculature. https://www.visualsonics.com/sites/default/files/AN_LAZR_NA_Vasculature_Structure_ver1.1.pdf.

[75] Sultan L.R., Grasso V., Jose J., Al-Hasani M., Karmacharya M.B., Sehgal C.M. (2024). Advanced Techniques for Liver Fibrosis Detection: Spectral Photoacoustic Imaging and Superpixel Photoacoustic Unmixing Analysis for Collagen Tracking. Sensors.

[76] Sun T., Lv J., Zhao X., Li W., Zhang Z., Nie L. (2023). In Vivo Liver Function Reserve Assessments in Alcoholic Liver Disease by Scalable Photoacoustic Imaging. Photoacoustics.

[77] Qiu Y., Li H., Yu K., Chen J., Qi L., Zhao Y., Nie L. (2025). Collagen Fibers Quantification for Liver Fibrosis Assessment Using Linear Dichroism Photoacoustic Microscopy. Photoacoustics.

[78] Deng D., Dai B., Wei J., Yuan X., Yang X., Qi S., Zhang Z. (2021). A Drawer-Type Abdominal Window with an Acrylic/Resin Coverslip Enables Long-Term Intravital Fluorescence/Photoacoustic Imaging of the Liver. Nanophotonics.

[79] Wei J., Zhang R., Jiang M., Gao L., Yu X., Liu X.L., Dai Y., Luo Q., Zhang Z., Yang X. (2025). Quantitative Longitudinal Investigation of Non-Alcoholic Steatohepatitis in Mice by Photoacoustic Microscopy. Photoacoustics.

[80] Zhou Q., Liu J., Li H., Qin A., Nie L. (2025). Optical Imaging Unveils Morphological Heterogeneity at the Hepatic Lobule Level during Metabolic Dysfunction-Associated Fatty Liver Disease Progression [Invited]. Biomed. Opt. Express..

[81] Li W., Lv J., Li H., Song L., Zhang R., Zhao X., Xuan F., Sun T., Long K., Zhao Y. (2025). Quantification of Vascular Remodeling and Sinusoidal Capillarization to Assess Liver Fibrosis with Photoacoustic Imaging. Radiology.

[82] Lautt W.W. (2009). Hepatic Circulation. Colloquium Series on Integrated Systems Physiology: From Molecule to Function.

[83] Imai T., Takahashi K., Goto F., Morishita Y. (1998). Measurement of Blood Concentration of Indocyanine Green by Pulse Dye Densitometry–Comparison with the Conventional Spectrophotometric Method. J. Clin. Monit. Comput..

[84] Qiu T., Yang J., Peng C., Xiang H., Huang L., Ling W., Luo Y. (2023). Diagnosis of Liver Fibrosis and Liver Function Reserve through Non-Invasive Multispectral Photoacoustic Imaging. Photoacoustics.

[85] Qiu T., Peng C., Huang L., Yang J., Ling W., Li J., Xiang H., Luo Y. (2023). ICG Clearance Test Based on Photoacoustic Imaging for Assessment of Human Liver Function Reserve: An Initial Clinical Study. Photoacoustics.

[86] Chen H., Li K., Yuan L., Zhang X.B. (2023). Design of a Near-Infrared Fluoro-Photoacoustic Probe for Rapid Imaging of Carboxylesterase in Liver Injury. Chem. Commun..

[87] Fan X., Ren T., Yang W., Zhang X., Yuan L. (2021). Activatable Photoacoustic/Fluorescent Dual-Modal Probe for Monitoring of Drug-Induced Liver Hypoxia: In Vivo. Chem. Commun..

[88] Yan Y.H., Zhou J.L., Ren L.L., Liang P.Z., Zhang W., Ren T.B., Yuan L., Yin X., Zhang X.B. (2025). An Activatable Fluorescence/Photoacoustic Bimodal Probe for the Detection of Drug-Induced Liver Senescence. Chemosensors.

[89] Wen D., Yan R., He J., Gu L., Chen X.Y., Wang K. (2025). Investigating the Efficacy of a Photoacoustic Probe in Liver Function Assessment for Diabetic Mellitus. Spectrochim. Acta A Mol. Biomol. Spectrosc..

[90] Wang K., Chen X.Y., Zhang R.W.Y., Yue Y., Wen X.L., Yang Y.S., Han C.Y., Ma Y., Liu H.J., Zhu H.L. (2024). Multifunctional Fluorescence/Photoacoustic Bimodal Imaging of γ-Glutamyltranspeptidase in Liver Disorders under Different Triggering Conditions. Biomaterials.

[91] Miao M., Miao J., Zhang Y., Zhang J., She M., Zhao M., Miao Q., Yang L., Zhou K., Li Q. (2023). An Activatable Near-Infrared Molecular Reporter for Fluoro-Photoacoustic Imaging of Liver Fibrosis. Biosens. Bioelectron..

[92] Liu H., Zhou Y., Gao H., Xu H., Liu Z. (2025). A Small-Molecular Photoacoustic Probe for Reversible and Ratiometric Imaging Oxidative Stress Dynamics in Acute Liver Injury Mice. Spectrochim. Acta A Mol. Biomol. Spectrosc..

[93] Liu X., Yang Y., Peng L., Li X., Qi F.Y., Zheng G., Tan L., Qiao L., Zhang C.H. (2025). A Superoxide Anion Activatable 7-Azaindole Derived Photoacoustic Probe for High-Contrast Imaging of Drug-Induced Liver Injury. Sens. Actuators B Chem..

[94] Wu H., Xia F., Zhang L., Fang C., Lee J., Gong L., Gao J., Ling D., Li F. (2022). A ROS-Sensitive Nanozyme-Augmented Photoacoustic Nanoprobe for Early Diagnosis and Therapy of Acute Liver Failure. Adv. Mater..

[95] Wu L., Zeng W., Ishigaki Y., Zhang J., Bai H., Harimoto T., Suzuki T., Ye D. (2022). A Ratiometric Photoacoustic Probe with a Reversible Response to Hydrogen Sulfide and Hydroxyl Radicals for Dynamic Imaging of Liver Inflammation. Angew. Chem. Int. Ed..

[96] Chen R., Li W., Li R., Ai S., Zhu H., Lin W. (2023). Cysteine-Activated Fluorescence/Photoacoustic Integrated Probe for Non-Invasive Diagnosis of Drug-Induced Liver Injury. Chin. Chem. Lett..

[97] Tan D., Xu L., Wang X., Lin W. (2024). An Activated Photoacoustic Probe for Visualization of CO during Drug-Induced Liver Injury and Repair. Microchem. J..

[98] Fan X., Wang H., Yang W., Ren T., Yuan L. (2023). Ratiometric Photoacoustic Imaging of Endogenous HNO In Vivo for Assessing Prodrug Release and Liver Injury. Chem. Commun..

[99] Liu L., Xu J., Zhang S., Chen H., Wang L., Shen X.C., Chen H. (2022). Rational Design of Dual Ratiometric Photoacoustic and Fluorescent Probe for Reliable Imaging and Quantitative Detection of Endogenous CO during Drug-Induced Liver Injury and Repair. Sens. Actuators B Chem..

[100] Xu J., Lv Z., Wang L., Wu X., Tan B., Shen X.C., Chen H. (2024). Tuning Tumor Targeting and Ratiometric Photoacoustic Imaging by Fine-Tuning Torsion Angle for Colorectal Liver Metastasis Diagnosis. Chem. Eur. J..

[101] Zhang E., Wang S., Zhang G., Li A., Kong R., Kong W., Zhao Y., Ju P., Qu F. (2024). High-Fidelity Imaging of Fatty Liver In Vivo with a Fluorescent and Photoacoustic Dual-Mode Probe by Taking Advantage of the Liver “First Pass Effect”. Microchem. J..

[102] Lucero M.Y., Tang Y., Zhang C.J., Su S., Forzano J.A., Garcia V., Huang X., Moreno D., Chan J. (2021). Activity-Based Photoacoustic Probe for Biopsy-Free Assessment of Copper in Murine Models of Wilson’s Disease and Liver Metastasis. Proc. Natl. Acad. Sci. USA.

[103] Zhu H., Li W., Ai S., Wan Y., Lin W. (2024). Novel Activated NIR-II Fluorescence/Ratio Photoacoustic Probe for Dual-Modality Accurate Imaging of Palladium Ions Overload in Mouse Liver. J. Hazard. Mater..

[104] Zhang H., Zeng S.L., Wu Y.Z., Zhang R.X., Liu L.J., Xue Q., Chen J.Q., Wong K.K.Y., Xu J.F., Ren Y.G. (2023). Handheld Photoacoustic Imaging of Indocyanine Green Clearance for Real-Time Quantitative Evaluation of Liver Reserve Function. Biomed. Opt. Express..

[105] Jiang Y., Pu K. (2017). Advanced Photoacoustic Imaging Applications of Near-Infrared Absorbing Organic Nanoparticles. Small.

[106] Arroyo J., Zhang J., Lediju Bell M.A. (2025). Motion-Based Dynamic Light Delivery to Minimize Laser-Related Thermal Damage While Preserving Photoacoustic Image Quality. J. Biomed. Opt..

[107] Li Q., Ali Z., Zakian C., di Pietro M., Honing J., O’Donovan M., Flisikowski K., Sarantos V., Pierre G., Gloriod J. (2026). Tethered Optoacoustic and Optical Coherence Tomography Capsule Endoscopy for Label-Free Assessment of Barrett’s Oesophageal Neoplasia. Nat. Biomed. Eng..

[108] Zhang K., Ge N., Shen L., Yang F., Qiu J., Guo J., Wang K., Wang S., Yang F., Sheng S. (2025). Recent Research Progress of Photoacoustic Endoscopy in the Digestive System. Endosc. Ultrasound..

[109] Yang J.J., Cho S.-W., Yao J. (2025). Eavesdrop at Clinical Depths: Deep Photoacoustic Imaging with Internal Light Illumination. Adv. Devices Instrum..

[110] Erfanzadeh M., Zhu Q. (2019). Photoacoustic Imaging with Low-Cost Sources; A Review. Photoacoustics.

[111] Tantu M.T., Farhana F.Z., Haque F., Koo K.M., Qiao L., Ross A.G., Shiddiky M.J.A. (2025). Pathophysiology, Noninvasive Diagnostics and Emerging Personalized Treatments for Metabolic Associated Liver Diseases. npj Gut Liver.

